# Culture-directed antibiotics in peritoneal dialysis solutions: a systematic review focused on stability and compatibility

**DOI:** 10.1007/s40620-023-01716-7

**Published:** 2023-08-07

**Authors:** Chau Wei Ling, Kamal Sud, Rahul Patel, Gregory Peterson, Troy Wanandy, Siang Fei Yeoh, Connie Van, Ronald Castelino

**Affiliations:** 1 Faculty of Medicine and Health, The University of Sydney, Camperdown, NSW 2006 Australia; 2 Nepean Kidney Research Centre, Department of Renal Medicine, Nepean Hospital, Sydney, NSW Australia; 3 Peritoneal Dialysis Unit, Regional Dialysis Centre, Blacktown Hospital, Sydney, NSW Australia; 4 School of Pharmacy and Pharmacology, University of Tasmania, Hobart, TAS Australia; 5 Department of Pharmacy, Royal Hobart Hospital, Hobart, TAS Australia; 6 Department of Clinical Immunology and Allergy, Royal Hobart Hospital, Hobart, TAS Australia; 7 Department of Pharmacy, National University Hospital, Singapore, Singapore; 8 Department of Pharmacy, Blacktown Hospital, Blacktown, NSW Australia

**Keywords:** Culture-directed antibiotics, Peritoneal dialysis solutions, Peritonitis, Stability

## Abstract

**Background:**

This systematic review summarises the stability of less commonly prescribed antibiotics in different peritoneal dialysis solutions that could be used for culture-directed therapy of peritonitis, which would be especially useful in regions with a high prevalence of multidrug antibiotic-resistant strains.

**Methods:**

A literature search of Medline, Scopus, Embase and Google Scholar for articles published from inception to 25 January, 2023 was conducted. Only antibiotic stability studies conducted in vitro and not recently reviewed by So et al*.* were included. The main outcomes were chemical, physical, antimicrobial and microbial stability. This protocol was registered in PROSPERO (registration number CRD42023393366).

**Results:**

We screened 1254 abstracts, and 28 articles were included in the study. In addition to those discussed in a recent systematic review (So et al., Clin Kidney J 15(6):1071–1078, 2022), we identified 18 antimicrobial agents. Of these, 9 have intraperitoneal dosing recommendations in the recent International Society for Peritoneal Dialysis (ISPD) peritonitis guidelines, and 7 of the 9 had stability data applicable to clinical practice. They were cefotaxime, ceftriaxone, daptomycin, ofloxacin, and teicoplanin in glucose-based solutions, tobramycin in Extraneal solution only and fosfomycin in Extraneal, Nutrineal, Physioneal 1.36% and 2.27% glucose solutions.

**Conclusions:**

Physicochemical stability has not been demonstrated for all antibiotics with intraperitoneal dosing recommendations in the ISPD peritonitis guidelines. Further studies are required to determine the stability of antibiotics, especially in icodextrin-based and low-glucose degradation products, pH-neutral solutions.

**Graphical abstract:**

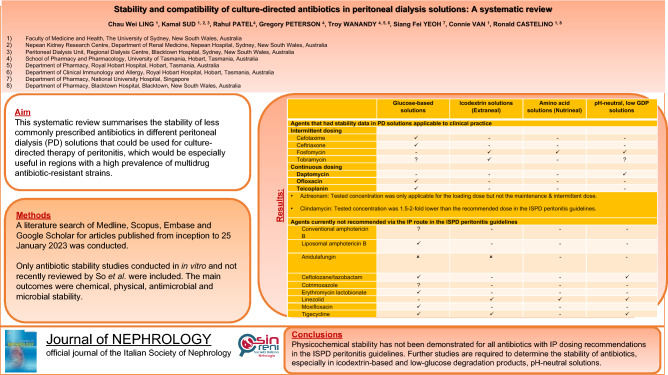

**Supplementary Information:**

The online version contains supplementary material available at 10.1007/s40620-023-01716-7.

## Introduction

Peritoneal dialysis (PD)-associated peritonitis remains a serious complication in patients receiving PD [1]. Repeated and prolonged episodes of peritonitis can result in structural alterations to the peritoneal membrane requiring permanent transfer to haemodialysis [2, 3]. Therefore, prompt treatment with intraperitoneal (IP) antibiotics admixed with PD solutions with a minimum dwell time of at least 6 h, as recommended by the International Society for Peritoneal Dialysis (ISPD) peritonitis guidelines [4], remains crucial for the treatment of PD-associated peritonitis.

The effective and safe treatment of peritonitis involves not only an appropriate choice and dosing of IP antibiotics, but also consideration of the stability of antibiotics in different PD solutions and their containers. So et al. [5] recently reviewed the stability and compatibility of commonly used antibiotics in PD solutions. These included penicillins, cephalosporins (cefazolin, ceftazidime, cefepime), vancomycin, gentamicin, ciprofloxacin, and carbapenems (imipenem, meropenem). However, information on the stability and compatibility of less commonly used antibiotics for the culture-directed treatment of PD-associated peritonitis in different PD solutions remains scarce, particularly in more recently developed neutral-pH and low-glucose degradation products (GDP) PD solutions that are marketed in multi-compartment bags [6]. While pharmacokinetic data are currently not available for many newer antibiotics for IP administration, the availability of data on the stability and compatibility of these antibiotics with PD solutions would facilitate immediate use when more pharmacokinetic information becomes available.

Therefore, our systematic review aimed to identify antibiotics not covered by So et al. [5] that are used for the culture-directed treatment of PD-associated peritonitis and provide a summary of their stability and compatibility in PD solutions, which would be especially useful to treat peritonitis in regions with a high prevalence of multidrug antibiotic-resistant strains. Furthermore, factors that need to be considered when examining the available stability data of IP antibiotics in PD solutions are discussed.

## Methods

### Search methodology

The reporting of this systematic review is in accordance with the Preferred Reporting Items for Systematic Reviews and Meta-Analyses (PRISMA). All articles (in English language) published from inception to 25 January, 2023 were identified from Medline, Scopus, Embase and Google Scholar. The following search terms were used: (stability) OR (compatibility) OR (“antimicrobial activity”) OR (“microbial growth”) OR (“microbial activity”) AND (“peritoneal dialysis fluid”) OR (“peritoneal dialysis solution”) OR (“dialysis fluid”) (Supplementary Material 1). This protocol was registered in PROSPERO (registration number CRD42023393366).

### Inclusion/exclusion criteria and study selection

The inclusion criteria were as follows: stability and compatibility studies conducted in vitro on antibiotics not covered by So et al. [5] and administered via the IP route for treatment of PD-associated peritonitis. The exclusion criteria were articles with drug stability not studied over time, types of PD solutions not specified or no longer in current use, and antibiotics that were reported by So et al. [5], i.e., penicillins (ampicillin, amoxicillin, piperacillin and tazobactam), cephalosporins (cefazolin, ceftazidime, cefepime), vancomycin, gentamicin, ciprofloxacin and carbapenems (imipenem, meropenem). The studies were assessed based on the following factors: studies that describe the materials used, test conditions and methods employed, types of analytical methods used, and studies that describe analytical determination at the outset (i.e., changes to the initial drug concentrations were determined from time-zero (starting point)) [7, 8].

### Definitions

Chemical, antimicrobial, physical and microbial stability of the IP antibiotics admixed in the PD solutions were assessed. Adequate chemical stability was defined as the initial drug concentration remaining > 90% from the time point when the antibiotic was added to the PD solutions and throughout the test period. Antimicrobial stability was defined as initial inhibitory zone diameters remaining > 90% from the time point when the antibiotic was added into the PD solutions and throughout the test period. Physical stability was defined as no signs of precipitation, the absence of particles visible through unaided vision and no discolouration of the antibiotics-PD solutions admixtures throughout the test period. Microbial stability was defined as the retention of the sterility of the PD solutions admixed with antibiotics throughout the test period.

The unmixed Physioneal and Balance PD solutions were defined as antibiotics added in the glucose compartment of the Physioneal PD solutions bag and the non-glucose compartment in the Balance PD solutions bag, respectively, as the injection port is only available in that compartment. The glucose compartment was kept separated from the non-glucose compartment during the storage period at refrigeration and/or room temperature.

The mixed Physioneal and Balance PD solutions were defined as antibiotics first added in the glucose compartment of the Physioneal PD solutions bag and the non-glucose compartment in the Balance PD solutions bag. The glucose compartment was then mixed with the non-glucose solutions in another compartment by breaking the intercompartment frangible pin or opening the middle seam between the two compartments to form the final solution immediately before warming the bag to body temperature (37 °C).

### Data extraction

The primary author (CWL) screened the abstracts of all articles and then reviewed the short-listed full-text articles for eligibility and relevance. The second reviewer (RLC) also independently reviewed all the articles short-listed by CWL. Any disagreements were resolved by discussion with the team. Table 1 summarises the variables of the included studies.

**Table 1:** Variables extracted from the studies included in the systematic review

The following data were extracted from the included studies:
1. Name of drug
2. Dosage recommendations on the ISPD peritonitis guidelines for the IP route
3. Author and year of publication
4. IP antibiotic concentration (in mg/L) administered in the PD bag
5. Name of PD solutions tested 6. Types of PD container material
7. Duration (in days or hours) and temperature of the antibiotic-PD solution admixtures that demonstrated stability
8. Outcomes (in terms of chemical, antimicrobial, physical and microbial stability)

*ISPD* International Society for Peritoneal Dialysis; *IP* intraperitoneal; *PD* peritoneal dialysis

## Results

### Selection of studies

**Fig. 1:**
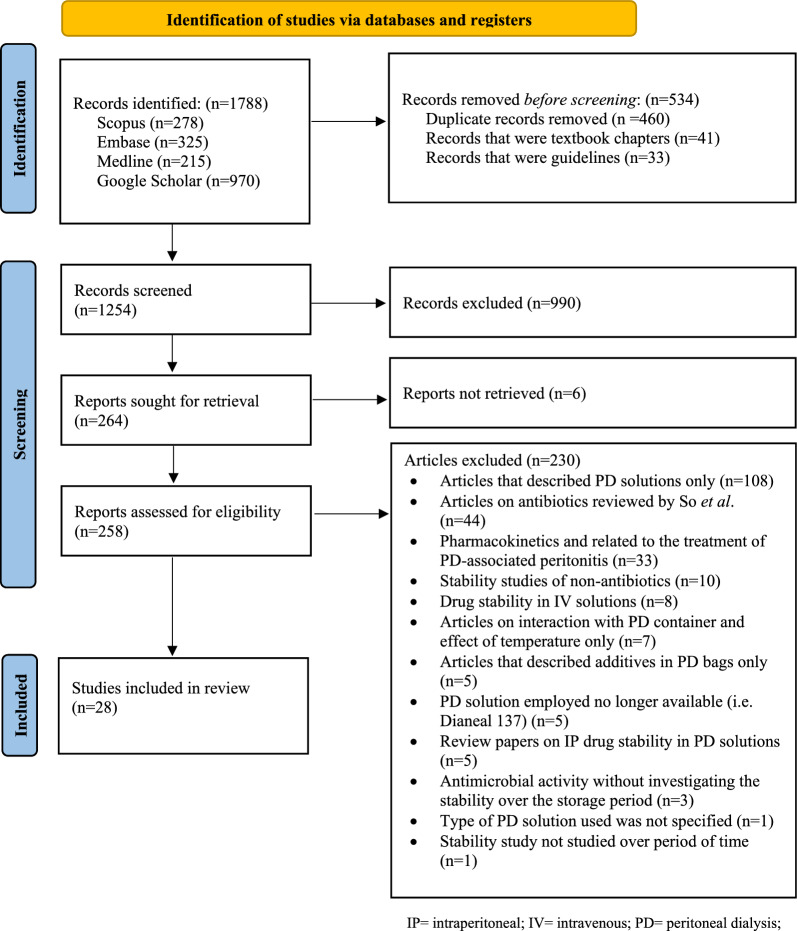
Search strategy and selection of studies

A total of 1788 articles were identified, and 1254 abstracts were screened for relevance after duplicates, textbook chapters and guidelines were removed. Two-hundred and fifty-eight articles were assessed for eligibility. A further 230 articles were excluded, and 28 were included in the final study (Fig. 1).

### IP antibiotics for culture-directed treatment in PD-associated peritonitis with stability and compatibility studies

**Fig. 2:**
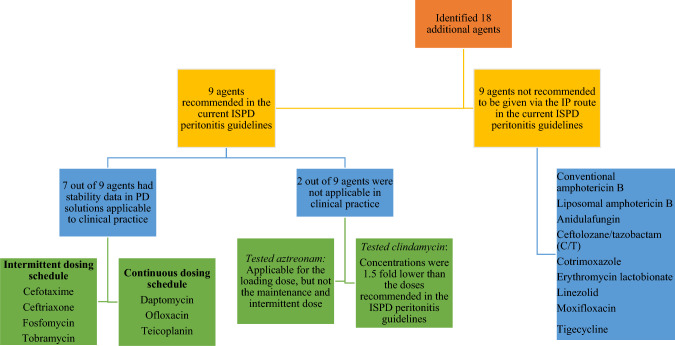
Summary of IP antibiotics for culture-directed treatment in PD-associated peritonitis with stability and compatibility studies

In addition to those discussed by So et al. [5], we identified 18 additional agents that can be employed as the culture-directed treatment for PD-associated peritonitis [4] (Supplementary Table 1 and Fig. 2). Of these, 9 had IP dosing recommendation in the recently released ISPD peritonitis guidelines [4]: aztreonam, cefotaxime, ceftriaxone, clindamycin, daptomycin, fosfomycin, ofloxacin, teicoplanin and tobramycin. However, only 7 of these 9 antibiotics had stability data in PD solutions applicable to clinical practice. They were cefotaxime, ceftriaxone, fosfomycin, and tobramycin administered in an intermittent dosing schedule, and daptomycin, ofloxacin and teicoplanin administered in continuous dosing. On the other hand, the published data on aztreonam and clindamycin were not applicable in clinical practice. First, the tested aztreonam concentration was only applicable for the loading dose but not the maintenance and intermittent dose. Second, the tested clindamycin concentrations were one-and-a-half-to-two-fold lower than doses recommended in the ISPD peritonitis guidelines [4].

Whilst the remaining 9 agents (conventional amphotericin B, liposomal amphotericin B, anidulafungin, ceftolozane/tazobactam (C/T), cotrimoxazole, erythromycin lactobionate, linezolid, moxifloxacin, and tigecycline) are not recommended to be given via the IP route in the current ISPD peritonitis guidelines [4], the stability information summarised in this review would enable their immediate use if clinically appropriate (Table 2).

**Table 2:** Summary of stability data for antibiotics in PD solutions

	Glucose-based solutions	Icodextrin solutions (Extraneal)	Amino acid solutions (Nutrineal)	pH-neutral, low GDP solutions
Agents that had stability data in PD solutions applicable to clinical practice				
Intermittent dosing				
Cefotaxime	✓^a^	–	–	–
Ceftriaxone	✓^b^	–	–	–
Fosfomycin	–	**	✓	✓^c^
Tobramycin	?	✓	-	?
Continuous dosing				
Daptomycin	–	–	–	✓^d^
Ofloxacin	✓^b^	–	–	–
Teicoplanin	✓^e^	–	–	–
Aztreonam: tested concentration was only applicable for the loading dose but not the maintenance and intermittent dose Clindamycin: tested concentration was 1.5–2-fold lower than the recommended dose in the ISPD peritonitis guidelines				
Agents currently not recommended via the IP route in the ISPD peritonitis guidelines				
Conventional amphotericin B	?	–	–	–
Liposomal amphotericin B	✓^f^	–	–	–
Anidulafungin	✗	✗	-	-
Ceftolozane/tazobactam	✓^g^	–	–	✓^h^
Cotrimoxazole	?	–	–	–
Erythromycin lactobionate	✓^i^	–	–	–
Linezolid	–	✓	✓	✓^j^
Moxifloxacin	✓^k^	–	–	–
Tigecycline	✓^l^	✓	–	✓^m^

^✓^ = Stable; ✗ = Unstable; −  = Not studied; ? = Cannot be extrapolated to clinical practice

^a^Dianeal PD-1 1.5% and 4.25% glucose, CAPD/DPCA ANDY disc 2 1.5% and CAPD/DPCA ANDY disc 4 2.3% glucose

^b^Dianeal PD 1.5% and 4.25% glucose solutions

^c^Mixed and unmixed Physioneal 1.36% and 2.27% glucose PD solutions

^d^Mixed and unmixed Balance 1.5% glucose solutions

^e^Dianeal PD-2 1.5% glucose solutions. However, findings were only applicable for maintenance dose of the continuous dosing (20 mg/L)

^f^ABLC 0.5 mg/L in Dianeal PD-1 1.5% glucose solutions, ABLC 2 mg/L in Dianeal PD-1 4.25% glucose solutions, ABLC 10 mg/L in Dianeal PD-1 1.5% glucose and 4.25% glucose solutions

^g^Dianeal 1.5% and 2.5% glucose solutions

^h^Unmixed and mixed Balance 1.3% and 2.3% glucose solutions; mixed and unmixed Physioneal 1.36%, 2.27% and 3.86% glucose solutions

^i^Dianeal PD-1 4.25% glucose solutions

^j^Mixed and unmixed Physioneal 40 1.36% and 2.27% glucose solutions

^k^Dianeal PD-1 1.36% and 3.86% glucose solutions

^l^Dianeal 1.5% glucose solutions

^m^Mixed and unmixed Balance 1.5% glucose solutions

## Clinical implications

The ISPD peritonitis guidelines provided dosage recommendations for antibiotics for treating PD-associated peritonitis. However, the stability and compatibility data on some of these antibiotics admixed in the PD solutions are limited [9–11].

The choice of PD solutions could vary across patients depending on their clinical needs [12, 13], centres' experience with particular PD solutions and their availability. Furthermore, different PD solutions vary in their electrolyte concentration, types of osmotic agents and buffers [14]. Therefore, data from a stability study in one type of PD solution cannot be extrapolated to another type of PD solution.

The stability data may influence the type of dosing schedule and choice of PD solutions in patients receiving IP antibiotics during peritonitis. In addition, for patients on continuous ambulatory peritoneal dialysis (CAPD), the typical regimen includes 4 exchanges with 6–8 h dwell time. On the other hand, patients on automated peritoneal dialysis (APD) have more frequent PD exchanges with variable dwell times from less than 1 h to up to 15 h. To ensure safe and effective treatment of peritonitis, it may be necessary to temporarily switch patients from APD with long dwells to CAPD to administer antibiotics that are stable for up to 6 h in PD solutions. However, this may be impractical due to the need to retrain patients (and carers) to perform CAPD, and a higher incidence of fluid overload [15] has been reported in patients on CAPD with peritonitis.

Furthermore, several antibiotics have demonstrated inferior drug stability when admixed with Physioneal solutions as compared to other PD solutions [16–18]. Possible explanations include drug interactions associated with complex formation from the buffering agent (bicarbonate/lactate) in Physioneal solutions and increased rate of drug degradation in alkaline PD solutions, such as lactate/bicarbonate and bicarbonate-based PD solutions (Physioneal and Bicavera) [19, 20]. Therefore, depending on the antibiotic's stability and compatibility data, patients may require a temporary change of PD solutions while receiving IP antibiotics for peritonitis. The antibiotics with stability and compatibility data for culture-directed treatment and their study limitations are discussed below.

### Amphotericin B and amphotericin B lipid complex

Prompt PD catheter removal and antifungal therapy for at least 2 weeks after catheter removal remains the cornerstone of treatment for fungal peritonitis [4]. However, immediate catheter removal may not be feasible, depending on the facilities, trained personnel to perform catheter removal and patients’ preferences. Therefore, bridging treatment with IP antifungal therapy while awaiting catheter removal is often required.

Amphotericin B is a parenteral polyene antifungal with a broad spectrum of activity against fungi (*Aspergillus* spp.,* Blastomyces dermatitidis*,* Paracoccidioides brasiliensis*,* Histoplasma capsulatum*, and *Coccidioides immitis*) [21, 22] and parasites (*Leishmania* spp.) [23]. Compared to the conventional amphotericin B formulation, the amphotericin B lipid complex (ABLC) formulations are associated with a lower incidence of nephrotoxicity [24] and infusion-related pain [25], but are more costly. Although IP conventional amphotericin B is uncommonly used nowadays, the availability of stability data will facilitate its use for treating fungal peritonitis [26] if treatment cost is a major consideration.

Janknegt et al. [27] evaluated the stability of conventional amphotericin B in concentrations of 1, 2 and 5 mg/L in glucose-based PD solutions (Dianeal 1.36% glucose) at body temperature (37 °C). At this temperature, all concentrations retained > 95% of initial drug concentrations for 6 h. However, as the drug stability with refrigeration was not assessed, the stability at refrigeration during storage remains unknown.

Manley et al. [28] investigated the stability of ABLC in concentrations of 0.5, 2 and 10 mg/L in glucose-based PD solutions (Dianeal PD-1 1.5% and 4.25% glucose) (Supplementary Table 1). To date, there are no clear dose recommendations for IP ABLC. Therefore, the dose of ABLC used in the stability study was likely derived from the pharmacokinetic data that achieved peak serum drug concentrations of amphotericin of 1.5–2.0 mg/L [29].

The authors noted yellow discolouration after ABLC was added to the PD solutions, although no visual changes were observed throughout the study period. This could be related to the natural physical form of amphotericin B, which appears yellow to orange. Nonetheless, as opposed to visual examination, further studies using sensitive analytical equipment such as ultraviolet–visible (UV–Vis) spectrophotometers are required to detect colour changes.

Based on the findings, ABLC 0.5 mg/L can be stored for up to 2 days at refrigeration and dwell for up to 6 h in Dianeal PD-1 1.5% glucose solution, while ABLC 2 mg/L is stable for up to 10 days at refrigeration and dwell for up to 2 days in Dianeal PD-1 4.25% glucose solutions. Amphotericin B lipid complex 10 mg/L can be stored for up to 14 days at refrigeration and dwell for up to 2 days in Dianeal PD-1 1.5% glucose and 4.25% glucose solutions. However, this study is limited by several issues that include (i) methodological and sample issues, such as the use of duplicate rather than triplicate sample analysis that might affect the margin of error, (ii) the use of a stability-indicating high-performance liquid chromatography (HPLC) technique in the analysis could not be verified (iii) inconsistency in the resuspension ability of ABLC samples as evidenced by a large standard deviation in some samples, and the (iv) decomposition of the lipid component of ABLC that was observed by the authors. Thus, further studies using improved methodologies are warranted in order to examine the implications of lipid complex degradation in amphotericin B stability and its effects on patient safety, efficacy and treatment tolerability before the findings can be extrapolated to clinical use.

### Anidulafungin

Anidulafungin is a parenteral semisynthetic echinocandin with broad antifungal activity against *Candida *spp. [30]. There is only one stability study available for anidulafungin. Tobudic et al. [31] evaluated the stability of anidulafungin 200 mg/L in glucose-based (Dianeal PD-4 1.36% glucose) and icodextrin-based (Extraneal 7.5%) solutions. At 4 and 25 °C, the authors demonstrated that anidulafungin retained > 95% drug concentrations for up to 14 days. At 36 °C, although anidulafungin retained > 90% initial concentrations in Extraneal 7.5% and Dianeal PD-4 1.36% glucose solutions, drug precipitation was observed. Possible explanations include microprecipitation from low pH in Dianeal and Extraneal solutions [32] and the high molecular weight of icodextrin in Extraneal [31, 33]. Therefore, the use of anidulafungin in these PD solutions is not recommended. Furthermore, the findings of this study must be interpreted with caution as the authors did not specify the formulation of anidulafungin employed in the stability study.^1^ The anidulafungin of the original formulation required dilution with 20% v/v dehydrated alcohol due to its poor aqueous solubility [34]. Thus, given the absence of data to support the long-term safety of alcohol in the peritoneum cavity, the findings may not be applicable in clinical practice (if the original anidulafungin formulation was employed in the study). Compared to the original formulation, the improved formulations contain polysorbate 80 to solubilise anidulafungin [35]. Thus, the findings from a stability study conducted with the original formulation of anidulafungin cannot be extrapolated to the new anidulafungin formulation, and vice versa.

### Aztreonam

Aztreonam is a monobactam antibiotic with good activity against gram-negative aerobic organisms. It is active against Enterobacteriaceae that are non-extended-spectrum beta-lactamases (ESBLs), *Klebsiella pneumoniae* carbapenemase (KPC)-type beta-lactamases or overproduction of AmpC beta-lactamases, such as *Escherichia coli, Proteus mirabilis, Klebsiella, Enterobacter, Serratia* and *Citrobacter* [36, 37], and could therefore be used as a culture-directed treatment of peritonitis caused by gram-negative organisms [38]. Tobudic et al*.* [39] investigated the stability of IP aztreonam in two concentrations (400 mg/L and 500 mg/L) in three different PD solutions: 500 mg/L in icodextrin-based solutions (Extraneal) and low GDP, pH-neutral (mixed and unmixed Physioneal 1.36 and 2.27% glucose), and 400 mg/L in amino acid-based solution (Nutrineal). At 37 °C, aztreonam was not stable in mixed Physioneal 1.36% and 2.27% glucose solutions, having a short period of stability of 4 h and 1 h, respectively. However, aztreonam was observed to be more stable in Nutrineal and Extraneal solutions. In Nutrineal solution, aztreonam retained > 90% of initial concentrations for 14 days at 6 °C, 7 days at 25 °C and 24 h at 37 °C. In Extraneal solution, aztreonam retained > 93% of initial concentrations for 14 days at 6 °C and 25 °C, and 24 h at 37 °C.

As the 400 mg/L tested concentration was not consistent with the loading dose required for continuous dosing (i.e., 500 mg/L), only data for aztreonam 500 mg/L as a loading dose admixed with Extraneal are applicable in clinical practice. Aztreonam 500 mg/L is stable at refrigeration for up to 14 days and dwell for up to 24 h in Extraneal 7.5% solution. Given the short duration of stability of aztreonam in mixed Physioneal 1.36% and 2.27% glucose solutions, it should not be administered in Physioneal PD solutions. Nevertheless, the tested concentration was lower than the intermittent dose (2 g once daily) recommended in the ISPD guidelines [4]; therefore, the findings for the intermittent dosing may not be applicable in clinical practice, and more stability studies are warranted to investigate the stability of aztreonam at higher concentrations.

### Cefotaxime

Cefotaxime is a third-generation extended-spectrum parenteral cephalosporin that exhibits excellent activity against many aerobic gram-negative bacilli but not *Bacteroides fragilis* [40] and *Pseudomonas aeruginosa* infections [40]. It has a spectrum of antimicrobial activity similar to ceftriaxone but requires multiple daily doses [41]. Cefotaxime has also been used successfully to treat PD-associated peritonitis caused by *Rhizobium radiobacter*, in combination with ciprofloxacin [42]. Three stability studies with cefotaxime were conducted in a glucose-based PD solution (Dianeal). Paap et al*.* [43] investigated the stability of IP cefotaxime 1000 mg/L (intermittent dose), which is the recommended dosage for the treatment of PD-associated peritonitis in the ISPD guidelines [4], admixed in Dianeal PD-1 1.5% and 4.25% glucose solutions. It was demonstrated that cefotaxime retained > 90% and 95% of initial concentrations for up to 24 h at 25 °C and 6 h at 37 °C, respectively, in both Dianeal PD-1 1.5% and 4.25% glucose solutions.

Sewell et al*.* [44] evaluated the antimicrobial activity of cefotaxime 125 mg/L in Dianeal PD-2 solutions containing heparin 500units/L. It was demonstrated that 95% antimicrobial activity was retained for 24 h at 25 °C, but a 15% loss of bioactivity was observed when stored at 48 h. However, there are two important points to note when considering these findings [44]. First, the concentration of cefotaxime employed in the study (125 mg/L) [44], was lower than the intermittent dose (500-1000 mg) recommended in the ISPD peritonitis guidelines [4]. Second, the authors did not specify the glucose concentration in Dianeal PD-2 solutions. Moreover, differences in pH were observed in solutions with varying glucose concentrations [45, 46]. Solutions containing higher glucose concentrations have demonstrated an accelerated rate of drug degradation [47, 48]. Therefore, the findings from Sewell et al. [44] cannot be extrapolated to all Dianeal PD-2 solutions containing varying glucose concentrations.

Fatooqi et al. [49] evaluated the stability of cefotaxime 1000 mg/L in a glucose-based PD solution (CAPD/DPCA ANDY disc 2 1.5% and CAPD/DPCA ANDY disc 4 2.3% glucose). In the studied PD solutions, it was demonstrated that cefotaxime retained > 90% drug concentrations for 7 days and 12 h at 4 °C and 37 °C, respectively. Additionally, the authors also evaluated the stability of cefotaxime at an elevated temperature of 40 °C, with a drug loss of up to 9.6% observed.

As the tested concentrations of cefotaxime by Paap and Nahata [43] and Fatooqi et al. [49] were consistent with the recommendations in the ISPD peritonitis guidelines [4], the findings for cefotaxime in Dianeal PD-1 1.5% and 4.25% glucose, CAPD/DPCA ANDY disc 2 1.5% and CAPD/DPCA ANDY disc 4 2.3% glucose can be extrapolated to clinical practice. Cefotaxime 1000 mg/L can be stored for up to 7 days at refrigeration and dwell for up to 12 h in CAPD/DPCA ANDY disc 2 1.5% and CAPD/DPCA ANDY disc 4 2.3% glucose solutions. In Dianeal PD-1 1.5% and 4.25% glucose, cefotaxime 1000 mg/L can dwell for up to 6 h. However, Paap and Nahata [43] did not evaluate the stability of cefotaxime at refrigeration. Thus, the stability of cefotaxime in Dianeal PD-1 1.5% and 4.25% glucose solutions during storage at refrigeration remains unknown.

### Ceftriaxone

Ceftriaxone is a third-generation cephalosporin active against many aerobic gram-negative bacilli and gram-positive organisms, including *Methicillin-susceptible Staphylococcus aureus* (MSSA), *Streptococcus pneumoniae* and *Neisseria meningitidis*. Intraperitoneal ceftriaxone 1 g administered once daily in one exchange [50], as recommended in the ISPD peritonitis guidelines [4], has been used successfully to treat peritonitis caused by *Pasteurella multocida* [51, 52].

There has been only one stability study available on ceftriaxone. Nahata et al. [53] investigated the stability of IP ceftriaxone 1000 mg/L in glucose-based PD solution (Dianeal PD-1 1.5% and 4.25% glucose). It was demonstrated that ceftriaxone administered as an intermittent dose retained > 90% initial concentrations for 14 days at 4 °C, 24 h at 23 °C, and 6 h at 37 °C in both PD solutions. The tested concentration was consistent with the ISPD peritonitis guideline recommendations [4], so IP ceftriaxone 1000 mg/L is stable for 14 days at refrigeration (4 °C). However, information on the stability of this antibiotic with dwell times longer than 6 h is currently unavailable for Dianeal PD-1 1.5% and 4.25% glucose solutions. Therefore, the dwell time of IP ceftriaxone 1000 mg/L should not exceed 6 h in Dianeal PD-1 1.5% and 4.25% glucose solutions.

### Ceftolozane/tazobactam

Ceftolozane and tazobactam (C/T) is a parenteral advanced-generation cephalosporin combined with a beta-lactamase inhibitor. It is effective against multidrug-resistant *Pseudomonas aeruginosa* infections [54, 55] and *Enterobacteriaceae* [56] that produce ESBLs.

Harmanjeet et al*.* [57] investigated the stability of IP C/T in three concentrations: 40/20 mg/L in low GDP, pH-neutral (unmixed Balance 1.3% and 2.3% glucose, 20/10 mg/L in mixed Balance 1.3% and 2.3% glucose, mixed Physioneal 1.36%, 2.27% and 3.86% glucose solutions) and glucose-based (Dianeal 1.5% and 2.5% glucose solutions), and 55.1/27.5 mg/L in unmixed Physioneal 1.36%, 2.27% and 3.86% glucose solutions. All tested C/T concentrations retained > 95% initial concentration for 7 days at 4 °C, 6 h at 25 °C and 12 h at 37 °C, in all studied PD solutions. The findings suggest that the tested C/T concentrations are stable for 7 days at refrigeration and can dwell for up to 12 h in all tested PD solutions.

### Clindamycin

Clindamycin is a lincosamide antibiotic and a derivative of lincomycin. It has good activity against aerobic gram-positive bacteria, particularly methicillin-resistant *Staphylococci* [58] and pneumococci, including penicillin-resistant *Streptococcus pneumoniae* [59]. Three stability studies on clindamycin were conducted in glucose-based PD solutions (Dianeal PD-2 and PD-4). Tran et al*.* [60] evaluated the stability of clindamycin 150 mg/L in Dianeal PD-4 solutions only at 37 °C and demonstrated stability for 6 h. Sewell et al*.* [44] evaluated the antimicrobial activity of clindamycin 10 mg/L with heparin 500units/L in Dianeal PD-2 solution and showed that > 99% of activity was retained for 2 days at 25 °C. Of note, Kohoe et al*.* [61] studied the stability of clindamycin 200 mg/L alone (Supplementary Table 1) and in combination with gentamicin in Dianeal PD-2 solution (Supplementary Table 2), and reported chemical stability when kept for 4 days at 8 °C and 23 °C. Although the authors stated there were no significant changes in the clindamycin concentrations over the 96-h test period, we must highlight that the authors reported an approximately 4% overfill of the PD bags during the study, resulting in inaccurate final drug concentrations [61]. Therefore, the results must be carefully interpreted when applying to clinical practice.

We note three important points to be considered with these findings. First, the tested clindamycin concentrations were 1.5–twofold lower than those recommended in the ISPD peritonitis guidelines [4], i.e., 300 mg/L administered in continuous dosing [4]. Second, the three studies only evaluated the stability of clindamycin at selected but clinically relevant storage temperatures. Third, the authors did not report the glucose concentration of Dianeal PD-2 and PD-4 solutions used in the study. Therefore, results from these stability data cannot be extrapolated to other glucose-based PD solutions with varying glucose concentrations.

### Cotrimoxazole

Cotrimoxazole (CoT) is the combination of trimethoprim and sulphamethoxazole in a fixed ratio of 1:5 that is active against penicillin-resistant and some fluoroquinolone-and methicillin-resistant strains of *Staphylococcus aureus* [62, 63], *Streptococcus saprophyticus* [64] and most of the coagulase-negative *Staphylococcus* spp. [65].

Holmes and Aldous [66] evaluated the stability of CoT in glucose-based solutions (Dianeal PD-2 4.25% glucose) stored in polyvinylchloride (PVC) containers and glass ampoules at 20 °C. The authors reported that CoT retained > 90% drug concentrations for up to 12 h when stored in PVC containers but up to 24 h in the glass ampoule. However, the findings must be interpreted with caution. Firstly, the authors did not evaluate the stability of CoT at refrigeration and body temperature. Thus, the stability of CoT during storage at refrigeration and dwell time remains unknown. Secondly, the CoT formulation (Bactrim^®^) employed in the study contained alcohol 12.7% v/v [67]. Therefore, the findings by Holmes and Aldous [66] cannot be applied in clinical practice. Further studies are warranted to evaluate the stability of a CoT alcohol-free formulation (i.e., Sevatrim^®^) in the PD solutions.

### Daptomycin

Daptomycin is a cyclic lipopeptide antibiotic that exhibits good bactericidal activity against gram-positive strains, including methicillin-resistant *Staphylococcus aureus* and vancomycin-resistant *enterococci* [68, 69]. Parra et al. [70] evaluated the stability of IP daptomycin (Cubicin^®^) 20 mg/L in neutral-pH PD solutions (mixed Physioneal 35) with two different glucose concentrations (1.36% and 2.27% glucose) held in a PVC container. In mixed Physioneal 35 1.36% and 2.27% glucose solution, daptomycin retained > 92% of the initial concentration for up to 24 h at 25 °C and 6 h at 37 °C. Compared to mixed Physioneal 35 1.36% glucose solutions, daptomycin was more stable in mixed Physioneal 2.27% glucose solutions at both room and body temperature. However, the stability study of daptomycin was not conducted in refrigerated conditions. Therefore, stability during storage at refrigeration remains to be determined. Additionally, Ramdas et al. [71] studied the stability of IP daptomycin (Cubicin^®^) in unmixed and mixed pH-neutral (Balance 1.5% glucose) PD solution. The authors found that daptomycin 25 mg/L in unmixed Balance 1.5% glucose solutions was stable for up to 5 days at 4 °C and 3 days at 25 °C and daptomycin 20 mg/L^2^ in mixed Balance 1.5% glucose solutions was stable for 12 h at 37 °C.

Peyro-Saint-Paul et al. [32] assessed the stability of IP daptomycin (Cubicin^®^) at 50 mg/L, 100 mg/L and 200 mg/L in low GDP, pH-neutral solution (Physioneal 40 1.36% glucose) and amino acid-based solution (Nutrineal) stored in PVC and glass containers. Compared to Physioneal 40 1.36% glucose solutions, the findings suggested that daptomycin 50 mg/L and 200 mg/L were more stable in Nutrineal solution stored in the PVC container at 4 °C and 25 °C, although > 90% of initial concentrations were retained in both PD solutions [32]. Nevertheless, the findings must be interpreted with caution. Firstly, the authors did not specify whether the stability study for daptomycin was administered in the unmixed or mixed Physioneal solutions. Secondly, although daptomycin 100 mg/L administered as a loading dose was stable for up to 7 days on refrigeration and dwell for up to 12 and 6 h when stored in the Physioneal 40 1.36% glucose and Nutrineal solutions, respectively, the studies were conducted with the PD solutions stored in a glass container. As Physioneal and Nutrineal solutions are only commercially available in PVC containers, the findings from Peyro-Saint Paul et al. [32] may not be applicable in clinical practice.

On the other hand, as daptomycin 20 mg/L as maintenance dose is consistent with the recommended dose in the ISPD peritonitis guidelines [4], the findings by Ramdas et al. [71] can be applied in clinical practice. Daptomycin 20 mg/L maintenance dose can be stored on refrigeration for up to 5 days and dwell for up to 12 h in mixed and unmixed Balance 1.5% glucose solutions.

Finally, it has to be highlighted that the stability data identified in this review for Cubicin^®^ (original formulation) must not be extrapolated to lyophilised daptomycin formulations (i.e., Cubicin RF^®^, Dapzura RT^®^, Daptomycin Hospira^®^). Compared to the original daptomycin [72], lyophilised daptomycin [73] contains sucrose and sodium hydroxide to increase stability at room temperature and facilitate reconstitution, respectively. Further studies are warranted to evaluate the stability of lyophilised daptomycin in PD solutions.

### Erythromycin lactobionate

Erythromycin is a broad-spectrum macrolide. It is active against gram-positive cocci such as *Staphylococcus aureus* and beta-lactamase-producing strains, coagulase-negative staphylococci, *Streptococcus pyogenes, and Streptococcus* spp. (*pneumoniae, viridans and bovis*) and some gram-negative bacteria such as *Moraxella catarrhalis, Legionella *spp., *Bordetella pertussis* and *Campylobacter jejuni* [74].

Kane et al. [75] evaluated the stability of IP erythromycin lactobionate 150 mg/L in glucose-based PD solutions (Dianeal PD-1 1.5% and 4.25% glucose). At 4 °C, erythromycin retained > 93% initial concentrations for 2 and 14 days in Dianeal PD-1 1.5% and 4.25% glucose solutions, respectively. At 25 °C, erythromycin retained ≥ 94% initial concentrations for up to 3 days in Dianeal PD-1 1.5% and 4.25% glucose solutions. However, at 37 °C, erythromycin retained > 90% drug concentrations for only 8 h in Dianeal PD-1 1.5% glucose solutions while up to 2 days in Dianeal PD-1 4.25% glucose PD solutions. Based on the findings, erythromycin 150 mg/L was more stable in Dianeal PD-1 4.25% glucose solutions than in Dianeal PD-1 1.5% glucose solutions. In Dianeal PD-1 1.5% glucose solutions, erythromycin can be stored for up to 2 days at refrigeration and dwell for up to 8 h. On the other hand, erythromycin can be stored for up to 14 days at refrigeration and dwell for up to 2 days in Dianeal PD-1 4.25% glucose solution.

### Fosfomycin

Fosfomycin is a phosphonic acid derivative, epoxide ring-containing and broad-spectrum antibiotic active against drug-resistant gram-negative and gram-positive microorganisms [76, 77]. Kussman et al. [78] conducted a stability study with three concentrations of IP fosfomycin: 1980 mg/L in icodextrin-based PD solution (Extraneal), 1587 mg/L in amino-acid based (Nutrineal), 5369 mg/L in the low GDP, neutral-pH (unmixed Physioneal 40 1.36% and 2.27% glucose), and 1980 mg/L in the mixed Physioneal 40 1.36% glucose solution. It was demonstrated that fosfomycin in all concentrations retained > 93% of initial concentration and > 95% antimicrobial activity for 14 days at 6 °C and 25 °C, and 24 h at 37 °C in all the PD solutions. Furthermore, the authors observed microbial stability of fosfomycin in all PD solutions when stored for 10 days at 37 °C. Based on the findings, the tested fosfomycin concentration that is consistent with the dose recommended in the ISPD peritonitis guidelines (i.e., 4 g as an intermittent dose in 2L PD bag) [4, 79] is stable for 14 days in Extraneal, Nutrineal, and mixed and unmixed Physioneal 1.36% and 2.27% glucose PD solutions when kept refrigerated and can be administered as a long-dwell for up to 24 h.

### Linezolid

Linezolid belongs to a newer oxazolidinone group and has been shown to be effective against organisms such as methicillin-resistant *S. epidermidis* (MRSE) and MRSA, vancomycin-resistant *Staphylococcus aureus* (VRSA) and VRE [80]. There were only two stability studies available for linezolid in PD solutions. Manley et al. [81] studied the stability of IP linezolid in concentrations of 150 mg/L, 300 mg/L and 600 mg/L in glucose-based PD solutions (Dianeal PD-2 1.5% and 4.25% glucose). It was demonstrated that linezolid at the three concentrations studied retained > 95% of initial concentrations for 7 days at 4 °C and 25 °C, and 24 h at 37 °C in both Dianeal PD solutions tested.

Poeppl et al. [82] studied the stability of IP linezolid in three concentrations: 214 mg/L in amino acid-based solution (Nutrineal), 260 mg/L in icodextrin-based solution (Extraneal) and in low GDP, neutral-pH solutions (mixed Physioneal 40 1.36% and 2.27% glucose solutions), and 585 mg/L in unmixed Physioneal 40 1.36% and 2.27% glucose. It was demonstrated that > 98% of initial concentrations remained for 14 days at 6 °C and 25 °C, and 24 h at 37 °C in all the tested PD solutions. In addition, linezolid retained > 95% antimicrobial activity in the tested PD solutions except in unmixed Physioneal 40 1.36% glucose PD solution, where 9.9% antimicrobial activity loss was observed when stored for 14 days at 6 °C. Based on the findings, all tested linezolid concentrations in all the PD solutions are stable with refrigeration for up to 14 days and can dwell for up to 24 h.

### Moxifloxacin

Moxifloxacin is a fourth-generation synthetic fluoroquinolone with activity against gram-positive bacteria. Although moxifloxacin has weaker activity against *Pseudomonas aeruginosa* strains [83], it is less susceptible to resistance from *Staphylococcus*, *Streptococcus pneumoniae* and *Escherichia coli* compared with ciprofloxacin [84, 85], while exhibiting better activity against *Stenotrophomonas maltophilia* [86, 87] than ciprofloxacin.

Fernandez-Varon et al*.* [88] investigated the stability of IP moxifloxacin 25 mg/L in two glucose-based PD solutions (Dianeal PD-1 1.36% and 3.86% glucose). In Dianeal PD-1 1.36% glucose solution, > 95% initial moxifloxacin concentration was retained for 14 days at 4 °C and 7 days at 25 °C. At 37 °C, moxifloxacin remained stable for up to 3 days in Dianeal PD-1 1.36% glucose solution. In Dianeal PD-1 3.86% glucose solution, moxifloxacin retained > 90% initial concentrations for 14 days at 4 °C and 3 days at 25 °C. However, at 37 °C, moxifloxacin was found to be stable for only 12 h [88]. Compared to Dianeal PD-1 3.86% glucose, moxifloxacin has longer stability in Dianeal PD-1 1.36% glucose solution at 37 °C (3 days versus 12 h), suggesting higher drug stability in PD solutions with a lower glucose concentration. Possible explanations for this observation could be glucose degradation which is related to the pH and has been associated with loss of drug potency, similar to that reported with daptomycin [89]. Based on the findings, IP moxifloxacin 25 mg/L is stable for up to 14 days at refrigeration in both Dianeal PD-1 1.36% and 3.86% glucose solutions. Moxifloxacin can dwell for up to 3 days in Dianeal PD-1 1.36% glucose solutions. However, until more data is available, moxifloxacin should not exceed a dwell time of 12 h in Dianeal PD-1 3.86% glucose solution.

### Ofloxacin

Ofloxacin is a second-generation fluoroquinolone antibiotic with broad-spectrum activity against aerobic gram-positive and gram-negative bacteria [90] and an add-on treatment for tuberculous peritonitis [91]. Although bactericidal concentrations of ofloxacin can be achieved with the oral route [92], the availability of appropriate stability data would enable its use when the IP route is indicated.

Battista et al*.* [93] investigated the stability of IP ofloxacin 25 mg/L in two glucose-based PD solutions (Dianeal PD-1 1.5% and 4.25% glucose). It was demonstrated that ofloxacin retained > 97% of its initial concentration for 14 days at 4 °C, 7 days at 25 °C and 48 h at 37 °C. The findings suggest that IP ofloxacin 25 mg/L, consistent with the maintenance dose of the continuous dosing recommended in the ISPD guidelines [4], is stable in Dianeal PD-1 1.5% and 4.25% glucose solutions for up to 14 days under refrigeration and can be allowed to dwell for up to 2 days at body temperature.

### Teicoplanin

Teicoplanin is a glycopeptide antibiotic structurally related to vancomycin [94]. It can be used as a reserved antibiotic to treat *Enterococcal faecalis* in patients with β-lactam allergy [95]. Teicoplanin with imipenem has been used successfully to treat PD-associated peritonitis caused by *Rhodococcus kroppenstedtii* [96]. There was only one stability study available for teicoplanin. Manduru et al. [97] evaluated the stability of IP teicoplanin 25 mg/L in a glucose-based solution (Dianeal PD-2 1.5% glucose). It was demonstrated that teicoplanin alone (Supplementary Table 1) or in combination with ceftazidime (Supplementary Table 2) retained > 90% initial concentrations at 4 °C for 7 days, followed by 16 h at 25 °C and 8 h at 37 °C in Dianeal PD-2 1.5% glucose solutions. However, there is an important point to be considered in the study. The tested teicoplanin concentration can only be extrapolated to the maintenance dose of the continuous dosing (20 mg/L) recommended in the ISPD peritonitis guidelines [4]. Further studies are required to evaluate the stability of a higher dose of teicoplanin in the PD solutions in patients requiring intermittent doses (15 mg/kg) or loading doses of the continuous dosing (400 mg/L) [4].

### Tigecycline

Tigecycline is the first glycylcycline antibiotic. It is active against multidrug-resistant strains, including several gram-positive (i.e., MRSA, VRE and penicillin-resistant *Streptococcus pneumoniae*) and gram-negative bacteria such as ESBL-producing *Enterobacteriaceae* and carbapenemase-resistant *Enterobacteriaceae* [98–100] owing to its ability to bind more strongly to the bacterial ribosome than tetracyclines. It is able to overcome organisms susceptible to tetracycline resistome [101, 102]. To date, only one stability study is available for tigecycline in PD solutions. Robiyanto et al*.* [103] investigated the stability of tigecycline at two concentrations: 2 mg/L in glucose-based solution (Dianeal 1.5% glucose), icodextrin-based solution (Extraneal) and low GDP, pH-neutral solution (mixed Balance 1.5% glucose), and 4 mg/L^3^ in unmixed Balance 1.5% glucose solutions. In Dianeal 1.5% glucose and Extraneal solutions, tigecycline 2 mg/L retained > 90% of the initial concentrations for 14 days at 4 °C, 3 days at 25 °C and 12 h at 37 °C. Conversely, in the unmixed Balance 1.5% glucose solution, tigecycline 4 mg/L remained stable for only up to 9 days at 4 °C and 3 days at 25 °C. At 37 °C, tigecycline 2 mg/L remained stable for up to 8 h in the mixed Balance 1.5% glucose solutions. Therefore, whilst the findings suggest that tigecycline retained > 90% initial concentrations across three different temperatures in all the tested PD solutions, tigecycline was more stable in Dianeal 2.5% glucose solution and Extraneal than in Balance 1.5% glucose PD solution [103]. In summary, tigecycline 2 mg/L can be stored on refrigeration for 14 days and dwell for up to 12 h in Dianeal 2.5% glucose and Extraneal PD solutions. On the other hand, tigecycline 4 mg/L can be stored in the unmixed Balance 1.5% glucose solutions at refrigeration for up to 9 days. After mixing the Balance 1.5% glucose solution, tigecycline 2 mg/L can dwell for up to 8 h.

### Tobramycin

Tobramycin is an aminoglycoside antibiotic that is more active against *Pseudomonas aeruginosa* and strains resistant to gentamicin [104]. There have been six stability studies conducted with varying concentrations of tobramycin. However, considering the recommended dose of 0.6 mg/kg daily, as in the ISPD peritonitis guidelines for the treatment of PD-associated peritonitis [4], only two studies are relevant to clinical practice and are discussed here.

Voges et al. [105] investigated the stability of IP tobramycin in two concentrations (78 mg/L and 60 mg/L) in three different PD solutions: 78 mg/L in low GDP, pH-neutral (mixed and unmixed Physioneal) and 60 mg/L in amino acid-based (Nutrineal), icodextrin-based (Extraneal) and glucose-based (Dianeal PD-4) solutions in non-PVC Clear Flex containers. When stored in the unmixed glucose compartment of Physioneal, tobramycin 78 mg/L retained > 99% of initial concentrations for 1 h at 25 °C, but lost 7% of the initial concentrations 1 h after the tobramycin was added into the glucose compartment of Physioneal, and just after mixing the PD bags at 25 °C [105]. In the mixed Physioneal PD solution, tobramycin had already lost 7.5% of its initial concentrations at the initial sampling time. A further loss of 16.3% and 20% of the initial concentrations were observed after 24 h at 25 °C and then a further 4 h of storage at 37 °C, respectively [105].

In Extraneal and Dianeal PD-4 solutions, tobramycin 60 mg/L retained > 93% of initial concentrations for 24 h at 25 °C. However, tobramycin lost > 10% of its initial concentration in Dianeal PD-4 solution when it was first stored for 24 h at 25 °C followed by another 4 h at 37 °C. Based on these findings, tobramycin 60 mg/L can be used in Nutrineal and Extraneal, but not in Dianeal and mixed Physioneal PD solutions, as > 10% loss of drug concentrations was observed in the latter solutions. However, there are two important points to be considered in this study. Firstly, the authors did not specify the glucose concentration in Physioneal and Dianeal PD-4 solutions studied. Therefore, data cannot be extrapolated to other Physioneal and Dianeal PD-4 solutions with varying glucose concentrations. Second, stability studies on storage under refrigeration were not conducted. Therefore, careful application of the results in clinical practice must be considered. Pallotta et al*.* [106] also looked into the stability of IP tobramycin 40 mg/L in an icodextrin-based PD solution (Extraneal). They demonstrated that ≥ 90% of the initial concentration was retained for 14 days at 4 °C, 7 days at 25 °C and up to 24 h at 37 °C. Based on the intermittent dose of 0.6 mg/kg [4] for patients weighing between 60 and 70 kg, as recommended in the ISPD peritonitis guidelines [4], only findings from Pallota et al. [106] are applicable in clinical practice. Thus, tobramycin 40 mg/L is stable under refrigeration (4 °C) for up to 14 days and can dwell for up to 24 h in Extraneal.

Furthermore, when combined with ceftazidime (Supplementary Table 2), Mason et al. [107] reported that both ceftazidime and tobramycin (125/8 mg/L) retained > 90% drug concentrations for up to 16 h at 25 °C followed by 8 h at 37 °C in Dianeal PD-2 2.5% glucose solutions. On the other hand, Deslandes et al. [18] reported that ceftazidime and tobramycin (125/4 mg/L) retained > 90% drug concentrations in low GDP, neutral pH solutions (mixed Physioneal 1.36% and 3.86% glucose) and icodextrin-based solutions (Extraneal), and that it was more stable in Extraneal solution (Supplementary Table 2). As Mason et al. [107] and Deslandes et al. [18] did not evaluate the stability of tobramycin and ceftazidime at refrigeration, the stability of this drug combination during storage at refrigeration is unknown. Furthermore, the findings for the stability of ceftazidime and tobramycin must be interpreted with caution as the dose of tobramycin employed in the study was nine-fold lower than the dose recommended in the ISPD peritonitis guidelines [4] (i.e. 0.6 mg/kg) for a patient weighing 60–70 kg.

### Study limitations

There were several limitations to this review. Firstly, the stability studies were conducted in in vitro settings. Thus, the clinical significance of the findings (particularly on the extent of drug loss from initial concentrations and PD container interactions) in practice is unknown. Secondly, the dose employed in the stability studies did not include drug concentrations of all dosing schedules. Therefore, the findings of one drug may not apply to its varying drug concentrations used in other dosing schedules. Thirdly, most of the studies identified in this review did not evaluate the stability of the antibiotic-PD solution admixture bag at various clinically relevant storage temperatures from when the antibiotic was first added to the PD bag until it is warmed prior to use, which mimics real-life practice. Fourthly, given that most studies employed visual examination to examine colour change and the presence of drug precipitation, subtle colour changes and microprecipitation of the antibiotic-PD admixtures cannot be excluded. Finally, whilst the lack of modern analytical methods i.e., stability-indicating HPLC, is the main limitation of the older stability studies, the existing data remain applicable in practice until more robust findings with stability-indicating methods become available. Nonetheless, the strength of this study lies in the ability to summarise the stability and compatibility of culture-directed antibiotics for the treatment of peritonitis in all available PD solutions through a systematic review.

### Other considerations for future stability studies

Several factors need to be considered when interpreting stability studies. These are discussed below.

1. Use of stability-indicating methods

The use of a stability-indicating method is crucial to differentiate whether the remaining drugs were from the intact drug or degradation products and other components. Of the 27 studies identified in this review, only 9 employed stability-indicating methods. The remaining studies were conducted using enzyme-multiplied immunoassay technique (EMIT), non-stability indicating liquid chromatography-mass spectrometry (LC–MS), spectrophotometric method and bioassays. Historically, EMIT and other immunoassays were commonly used in hospital laboratories and pharmaceutical stability studies [108]. Despite its selectivity, the immunoassay analytical technique may not be specific to the intact drug compared to the stability-indicating assays. The antibody in immunoassays may cross-react with degradation products that are chemically and structurally similar to the parent component, resulting in misleading results [109]. We therefore highlight the importance of using proven stability-indicating methods in all future stability studies.

(B) Employment of analytical techniques to determine physical incompatibilities

Of the 27 studies identified in this review, only 1 employed light microscopy to detect drug precipitation in the stability study [57]. Although visual examination is associated with lower costs, it cannot detect microprecipitation and subtle colour changes. Furthermore, visual examination of colour changes is dependent on an individual’s colour perception and is susceptible to perceptual bias. Therefore, future stability studies should consider employing analytical techniques (e.g. light microscopy, UV–Vis spectrophotometer, and nephelometer) to determine early signs of physical incompatibilities, such as subtle changes in colour and the presence of microprecipitation.

(C) Employment of study designs relevant to clinical practice

Most stability studies were conducted with three sets of PD bag samples, and each bag was exposed to various temperatures: refrigerated (4–8 °C), room (20–25 °C) and body (35–37 °C) temperatures. Of the 27 stability studies identified in this review, two studies exposed the same PD bag across various temperatures [18, 105]. However, they were only tested at both room (23 °C and 25 °C) and body temperature (37 °C). Therefore, the extent of drug loss from all time points and storage temperatures cannot be accurately determined. Only one study [97] determined the stability of the antibiotic-PD admixtures at various clinically relevant storage temperatures from when the antibiotic was first administered to the PD bag until it was warmed prior to use which mimics real-life practice.

Next, antibiotic-PD admixtures may be subjected to temperature variability during transportation (i.e., from the hospital to patients’ homes). Contrary to the environment in the pharmaceutical laboratory with constant temperature monitoring systems, the effects of temperature fluctuations and the duration outside refrigerated conditions during transportation on drug stability remain unknown. Data on the best practice for warming the PD bag prior to use remains unclear [110]. Whilst the manufacturers have recommended heating PD solutions with dry heat (i.e., heating pad), it must be noted that overheating could result in drug degradation and the formation of toxic GDP [110, 111]. Therefore, the accidental overheating of the PD bag beyond 37 °C in the patient’s home environment, which could potentially affect the drug stability resulting in loss of pharmacological activity, cannot be ruled out.

(D) Addition of heparin to the PD bag admixed with antibiotics

Heparin is commonly admixed to the PD bag to prevent PD catheter occlusion and dissolve fibrin clots [112]. Although a few stability studies have shown a negligible effect of heparin on the stability of IP antibiotics in the PD solutions [44, 113, 114], data cannot be extrapolated to other PD solutions considering the variable composition in each PD solution and chemical characteristics of each antibiotic. Moreover, the stability of heparin itself can be influenced by the pH of the PD solution admixed with antibiotics [115], storage conditions, and temperature [116]. Incompatibility of heparin and the PD solution admixed with the antibiotics can lead to chemical and physical degradation of heparin, resulting in the loss of anticoagulant activity [117] and precipitation [118].

(E) Use of a bioassay as a sole method to determine the stability

Historically, the microbiological method (i.e., standard disk diffusion) was commonly employed to evaluate drug stability in PD solutions [119]. In our review, we found that two studies [44, 120] only employed bioassays to evaluate drug stability. Although the information on the extent of antimicrobial activity remains imperative to ensure the effective treatment of PD-associated peritonitis, the use of a microbiological assay alone cannot accurately differentiate whether the antimicrobial activity is from the intact drug or drug degradation products [119]. Moreover, the antibiotic potency based on the inhibitory effect on the reference bacteria in the bioassays may not be extrapolated to other organisms with higher minimum inhibitory concentrations compared to the tested organisms.

(F) Microbial stability of the PD solutions

As antibiotics-supplemented PD bags are often pre-prepared in bulk for patients with PD-associated peritonitis managed in outpatient settings, it is imperative to ensure the sterility of the resultant products. Moreover, PD solutions do not contain bacteriostatic or antimicrobial agents. Antibiotic-PD admixtures are prepared by injecting the antibiotics into the PD bag via the injection port aseptically. Generally, the shelf-life of injectable antibiotics is limited to 24 h after reconstitution [121]. However, the antibiotic-PD admixtures are commonly stored over a few days and data on the microbial stability in admixtures are scarce. In this review, only one study [78] evaluated the microbial stability of the PD solutions across various temperatures and their duration at storage. Therefore, we propose that future studies should also consider microbial stability when conducting stability studies to ensure the sterility of the antibiotic-PD admixtures are maintained throughout the entire storage duration to the time of administration.

(G) PD container interactions

The types of material making up the PD bag can significantly affect the stability and compatibility of IP antibiotics due to the degree of drug adsorption to the container [119]. Of note, several studies demonstrated a loss of drug potency by adsorption when stored in PVC containers compared to glass bottles and non-PVC Clear Flex containers [122–124]. Interestingly, one of the studies [57] included in the review found negligible drug adsorption between various containers, such as polyolefin in Balance PD solution, and PVC in Dianeal and Physioneal PD solutions. However, drug adsorption to the PD container is multifactorial [125] and is dependent on various factors, including duration and temperature during storage, the pH of the PD solutions and its admixture with antibiotics, and the composition of the PD solutions. Therefore, more physicochemical stability studies with different antibiotics in various PD solutions and their containers are warranted.

(H) Use of replicate samples in study designs

To minimise the risk of human errors and assay variability [7], a minimum of three replicated samples should be prepared for quality control purposes [126]. In this review, all the studies employed triplicate samples, except for Mason et al. [107], Manley et al. [81], and Holmes and Aldous [66]. The authors had only prepared duplicated samples which could lead to a margin of errors in the results. Therefore, we emphasise the importance of three replicated samples in future studies to provide more reliable and robust results.

## Conclusion

The effective treatment of PD-associated peritonitis involves consideration of the appropriate antibiotic, its dose and dosing schedule based on pharmacokinetic data, and the stability and compatibility of IP antibiotics in the PD solution and its container. Given that the physicochemical behaviour of drugs varies across different PD solutions, stability data cannot be extrapolated from one PD solution to another. Among antibiotics that are used for the culture-directed treatment of peritonitis with IP dosing recommendations in the ISPD peritonitis guidelines (4), stability has only been demonstrated for cefotaxime, ceftriaxone, daptomycin, ofloxacin, and teicoplanin in glucose-based solutions, tobramycin in Extraneal solution only, and fosfomycin in Extraneal, Nutrineal, and Physioneal 1.36% and 2.27% glucose solutions. However, the stability data for the drugs summarised in this review cannot be extrapolated to the same drug with different concentrations.

With the increasing evidence pointing towards the use of neutral-pH and low-GDP PD solutions, more stability studies conducted on these solutions are warranted to ensure safe and effective treatment outcomes in PD-associated peritonitis. Future stability studies should adhere to the following recommended methodological approach: (i) using stability-indicating assays, (ii) conducting studies in clinically relevant storage conditions that include varying temperatures and durations of storage, (iii) investigating clinically relevant antibiotic concentrations for different dosing schedules and in different types of available PD solutions, (iv) assessing microbial stability to provide clinically valuable outcomes.

### Supplementary Information

Below is the link to the electronic supplementary material.

Supplementary file1 (DOCX 23 kb)

Supplementary file2 (DOCX 165 kb)

## Data Availability

Data that are relevant to the systematic review are available as online supplementary information.

## Footnotes

1 Unable to contact the corresponding author of Tobudic et al. to verify the type of anidulafungin formulation used in the study.

2 Daptomycin’s final concentration after mixing PD solutions from the two compartments (glucose and non-glucose solutions) in Balance 1.5% glucose solutions before warming to 37 °C on a heating plate.

3 Final concentration will be 2 mg/L after mixing solutions from the non-glucose and glucose compartments together.
