# Supplementary material for: Culture-directed antibiotics in peritoneal dialysis solutions: a systematic review focused on stability and compatibility
Source: J Nephrol. 2023 Aug 7;36(7):1841–59. doi: 10.1007/s40620-023-01716-7 (PMC10543841; doi:10.1007/s40620-023-01716-7)
Supplement: Supplementary file 1 — Supplementary file1 (DOCX 23 kb) [file 40620_2023_1716_MOESM1_ESM.docx]

**Stability and compatibility of culture-directed antibiotics in peritoneal dialysis solutions: A systematic review**

**Chau Wei LING ^1^, Kamal SUD ^1,^ ^2, 3^, Rahul P. PATEL^4^, Gregory M. PETERSON ^4^, Troy WANANDY ^4, 5, 6^, Siang Fei YEOH ^7^, Connie VAN ^1^, Ronald L. CASTELINO ^1, 8^**

1. Faculty of Medicine and Health, The University of Sydney, New South Wales, Australia
2. Nepean Kidney Research Centre, Department of Renal Medicine, Nepean Hospital, Sydney, New South Wales, Australia
3. Peritoneal Dialysis Unit, Regional Dialysis Centre, Blacktown Hospital, Sydney, New South Wales, Australia
4. School of Pharmacy and Pharmacology, University of Tasmania, Hobart, Tasmania, Australia
5. Department of Pharmacy, Royal Hobart Hospital, Hobart, Tasmania, Australia
6. Department of Clinical Immunology and Allergy, Royal Hobart Hospital, Hobart, Tasmania, Australia
7. Department of Pharmacy, National University Hospital, Singapore
8. Department of Pharmacy, Blacktown Hospital, Blacktown, New South Wales, Australia

**Scopus**

( TITLE-ABS-KEY ( stability )  OR  TITLE-ABS-KEY ( compatibility )  OR  TITLE-ABS-KEY ( "antimicrobial activity" )  OR  TITLE-ABS-KEY ( "microbial growth" )  OR  TITLE-ABS-KEY ( "microbial activity" )  AND  TITLE-ABS-KEY ( "peritoneal dialysis fluid" )  OR  TITLE-ABS-KEY ( "peritoneal dialysis solution" )  OR  TITLE-ABS-KEY ( "dialysis fluid" ) )

**Embase**

| 1 | Drug stability/or stability.mp |
| --- | --- |
| 2 | Compatibility/mp. |
| 3 | Antimicrobial activity.mp or antimicrobial activity/ |
| 4 | Microbial growth.mp. |
| 5 | Dialysis fluid/or peritoneal dialysis fluid/or peritoneal dialysis solution.mp. |
| 6 | 1 or 2 or 3 or 4 |
| 7 | 5 and 6 |

**Medline**

| 1 | Stability.mp. or Drug Stability/ |
| --- | --- |
| 2 | Compatibility.mp. |
| 3 | Antimicrobial activity.mp or antimicrobial activity/ |
| 4 | Microbial growth.mp. |
| 5 | Dialysis Solutions/or peritoneal dialysis solution.mp. |
| 6 | Dialysis fluid.mp. |
| 7 | 1 or 2 or 3 or 4 |
| 8 | 5 or 6 |
| 9 | 7 and 8 |

**Google scholar**

(("stability" OR "compatibility") AND ("peritoneal dialysis solution"))
