# Supplementary material for: Culture-directed antibiotics in peritoneal dialysis solutions: a systematic review focused on stability and compatibility
Source: J Nephrol. 2023 Aug 7;36(7):1841–59. doi: 10.1007/s40620-023-01716-7 (PMC10543841; doi:10.1007/s40620-023-01716-7)
Supplement: Supplementary file 2 — Supplementary file2 (DOCX 165 kb) [file 40620_2023_1716_MOESM2_ESM.docx]

**Stability and compatibility of culture-directed antibiotics in peritoneal dialysis solutions: A systematic review**

**Chau Wei LING ^1^, Kamal SUD ^1,^ ^2, 3^, Rahul P PATEL^4^, Gregory M PETERSON ^4^, Troy WANANDY ^4, 5, 6^, Siang Fei YEOH ^7^, Connie VAN ^1^, Ronald L CASTELINO ^1, 8^**

1. Faculty of Medicine and Health, The University of Sydney, New South Wales, Australia
2. Nepean Kidney Research Centre, Department of Renal Medicine, Nepean Hospital, Sydney, New South Wales, Australia
3. Peritoneal Dialysis Unit, Regional Dialysis Centre, Blacktown Hospital, Sydney, New South Wales, Australia
4. School of Pharmacy and Pharmacology, University of Tasmania, Hobart, Tasmania, Australia
5. Department of Pharmacy, Royal Hobart Hospital, Hobart, Tasmania, Australia
6. Department of Clinical Immunology and Allergy, Royal Hobart Hospital, Hobart, Tasmania, Australia
7. Department of Pharmacy, National University Hospital, Singapore
8. Department of Pharmacy, Blacktown Hospital, Blacktown, New South Wales, Australia

**Supplementary Table 1**: Summary of stability data of culture-directed antibiotics in PD solutions

| **Drug** | **Dosing recommendations on ISPD guidelines for IP route** | **Author/Year** | **Concentration (mg/L)^a^** | **PD Solution** | **PD container material** | **Temperature**  **(°C)** | **Stability in Days (d)/Hours (h)** | **Assay** | **Outcomes** | | | |
| --- | --- | --- | --- | --- | --- | --- | --- | --- | --- | --- | --- | --- |
|  |  |  |  |  |  |  |  |  | **Chemical Stability**  **(Initial concentration remained/%)** | **Antimicrobial activity (%)** | **Physical Stability** | **Microbial Stability** |
| Amphotericin B | N/A | Janknegt *et al*.^(1)^/1990 | 1 | Dianeal 1.36% glucose | Glass | 37 | 6h | HPLC | 96 | Not studied | No visual changes to the colour or precipitation | Not studied |
|  |  |  | 2 | Dianeal 1.36% glucose | Glass |  | 6h |  | 105 |  |  |  |
|  |  |  | 5 | Dianeal 1.36% glucose | Glass |  | 6h |  | 99 |  |  |  |
| Amphotericin B lipid complex | N/A | Manley *et al*.^(2)^/2000 | 0.5 | Dianeal PD-1 1.5% glucose | PVC | 4 | 2d | HPLC | 90.7 | Not studied | Yellow discolouration was observed after amphotericin B lipid complex was added into the PD solutions, but no visual changes throughout the period | Not studied |
|  |  |  |  |  |  | 25 | <24h |  | 77.8 |  |  |  |
|  |  |  |  |  |  | 37 | 6h |  | 91.7 |  |  |  |
|  |  |  |  | Dianeal PD-1 4.25% glucose | PVC | 4 | 2d |  | 90.3 |  |  |  |
|  |  |  |  |  |  | 25 | <24h |  | 89.3 |  |  |  |
|  |  |  |  |  |  | 37 | 1h |  | 91.8 |  |  |  |
|  |  |  | 2.0 | Dianeal PD-1 1.5% glucose | PVC | 4 | <24h |  | 76.2 |  |  |  |
|  |  |  |  |  |  | 25 | <24h |  | 85.8 |  |  |  |
|  |  |  |  |  |  | 37 | 2d |  | 99.3 |  |  |  |
|  |  |  |  | Dianeal PD-1 4.25% glucose | PVC | 4 | 10d |  | 91.7 |  |  |  |
|  |  |  |  |  |  | 25 | 24h |  | 90.7 |  |  |  |
|  |  |  |  |  |  | 37 | 2d |  | 96.9 |  |  |  |
|  |  |  | 10 | Dianeal PD-1 1.5% glucose | PVC | 4 | 14d |  | 91.8 |  |  |  |
|  |  |  |  |  |  | 25 | 14d |  | 96.6 |  |  |  |
|  |  |  |  |  |  | 37 | 2d |  | 104 |  |  |  |
|  |  |  |  | Dianeal PD-1 4.25% glucose | PVC | 4 | 14d |  | 96.8 |  |  |  |
|  |  |  |  |  |  | 25 | 14d |  | 96.8 |  |  |  |
|  |  |  |  |  |  | 37 | 2d |  | 101.7 |  |  |  |
| Anidulafungin | N/A | Tobudic *et al*.^(3)^/2014 | 200 | Dianeal PD-4 1.36% glucose | PVC | 4 | 14d | HPLC | 98.1 | Not studied | No visual changes to the colour but precipitation was observed at 36°C | Not studied |
|  |  |  |  |  |  | 25 | 14d |  | 100.1 |  |  |  |
|  |  |  |  |  |  | 36 | 14d |  | 91.3 |  |  |  |
|  |  |  |  | Extraneal 7.5% icodextrin | PVC | 4 | 14d |  | 96.9 |  |  |  |
|  |  |  |  |  |  | 25 | 14d |  | 100.0 |  |  |  |
|  |  |  |  |  |  | 36 | 14d |  | 96.8 |  |  |  |

IP=intraperitoneal; HPLC= high performance liquid chromatography; PVC= polyvinyl chloride

| **Drug** | **Dosing recommendations on ISPD guidelines for IP route** | **Author/Year** | **Concentration (mg/L)** | **PD Solution** | **PD container material** | **Temperature**  **(°C)** | **Stability in Days (d)/Hours (h)** | **Assay** | **Outcome(s)** | | | |
| --- | --- | --- | --- | --- | --- | --- | --- | --- | --- | --- | --- | --- |
|  |  |  |  |  |  |  |  |  | **Chemical Stability**  **(Initial concentration remained/%)** | **Antimicrobial activity (%)** | **Physical Stability** | **Microbial Stability** |
| Aztreonam | **Intermittent dose**:  2g/day  **Continuous dosing:** Loading dose: 500 mg/L Maintenance dose:  250 mg/L^(4)^ | Tobudic *et al*.^(5)^/2020 | 500 | Extraneal 7.5% icodextrin | PVC | 6 | 14d | HPLC ^c^/Disk diffusion inhibition assay with *Escherichia coli* | 98.5 | 99.2 | No visual changes to the colour or precipitation | Not reported |
|  |  |  |  |  |  | 25 | 14d |  | 93.2 | 100.3 |  |  |
|  |  |  |  |  |  | 37 | 24h |  | 96.2 | 102.2 |  |  |
|  |  |  |  | Physioneal 1.36% glucose | PVC | 6^[[1]](#footnote-1)^ | 14d |  | 96.8 | 97.3 |  |  |
|  |  |  |  |  |  | 25^a^ | 7d |  | 90.5 | 100.6 |  |  |
|  |  |  |  |  |  | 37^[[2]](#footnote-2)^ | 4h |  | 90.0 | 97.8 |  |  |
|  |  |  |  | Physioneal 2.27% glucose | PVC | 6 ^a^ | 14d |  | 94.4 | 100.5 |  |  |
|  |  |  |  |  |  | 25 ^a^ | 3d |  | 92.1 | 93.4 |  |  |
|  |  |  |  |  |  | 37 ^b^ | 1h |  | 93.4 | 113.8 |  |  |
|  |  |  | 400 | Nutrineal | PVC | 6 | 14d |  | 92.7 | 109.2 |  |  |
|  |  |  |  |  |  | 25 | 7d |  | 91.3 | 92.6 |  |  |
|  |  |  |  |  |  | 37 | 24h |  | 94.7 | 91.4 |  |  |
| Cefotaxime | **Intermittent dosing**:  500-1000^*^ mg/day ^(6)^  **Continuous dosing:**  N/A  ^*^_Pharmacokinetic study was conducted with IP cefotaxime 1g/L_ | Paap and Nahata.^(7)^/1990 | 1000 | Dianeal PD-1 1.5% glucose | PVC | 25 | 24h | HPLC ^[[3]](#footnote-3)^ | 92.4 | Not studied | No visual changes to the colour or precipitation | Not studied |
|  |  |  |  | Dianeal PD-1 4.25% glucose |  |  | 24h |  | 92.0 |  |  |  |
|  |  |  |  | Dianeal PD-1 1.5% glucose |  | 37 | 6h |  | 97.9 |  |  |  |
|  |  |  |  | Dianeal PD-1 4.25% glucose |  |  | 6h |  | 96.4 |  |  |  |
|  |  | Sewell *et al*.^(8)^/1983 | 125 | Dianeal PD-2 ^[[4]](#footnote-4)^ ^[[5]](#footnote-5)^ | PVC | 25 | 24h | Standard disk diffusion assay with *Bacillus subtilis* | Not studied | 95 | Not studied | Not studied |
|  |  | Fatooqi *et al*.^(9)^/2013 | 1000 | CAPD/DPCA ANDY disc 2  1.5% glucose | PVC | 4 | 7d | Spectrophotometric method | 98.2 | Not studied | Yellow discolouration was observed in all samples except in the refrigerated solution | Not studied |
|  |  |  |  |  |  | 24 | 12h |  | 94.4 |  |  |  |
|  |  |  |  |  |  | 37 | 12h |  | 91.0 |  |  |  |
|  |  |  |  |  |  | 40 | 8h |  | 90.3 |  |  |  |
|  |  |  | 1000 | CAPD/DPCA ANDY disc 4  2.3% glucose | PVC | 4 | 7d |  | 98.6 | Not studied |  | Not studied |
|  |  |  |  |  |  | 24 | 12h |  | 95.7 |  |  |  |
|  |  |  |  |  |  | 37 | 12h |  | 93.1 |  |  |  |
|  |  |  |  |  |  | 40 | 8h |  | 90.4 |  |  |  |

IP=intraperitoneal; HPLC= high performance liquid chromatography; PVC= polyvinyl chloride

| **Drug** | **Dosing recommendations on ISPD guidelines for IP route** | **Author/Year** | **Concentration (mg/L)^a^** | **PD Solution** | **PD container material** | **Temperature**  **(°C)** | **Stability in Days (d)/Hours (h)** | **Assay** | **Outcome(s)** | | | |
| --- | --- | --- | --- | --- | --- | --- | --- | --- | --- | --- | --- | --- |
|  |  |  |  |  |  |  |  |  | **Chemical Stability**  **(Initial concentration remained/%)** | **Antimicrobial activity (%)** | **Physical Stability** | **Microbial Stability** |
| Ceftriaxone | **Intermittent dosing:**  1000 mg/L/day ^(10)^ | Nahata *et al*.^(11)^/1991 | 1000 | Dianeal PD-1 1.5% glucose | PVC | 4 | 14d | HPLC c | 93.2 | Not studied | Not reported | Not studied |
|  |  |  |  |  |  | 23 | 24h |  | 91.0 |  |  |  |
|  |  |  |  |  |  | 37 | 6h |  | 93.0 |  |  |  |
|  |  |  |  | Dianeal PD-1 4.25% glucose |  | 4 | 14d |  | 92.7 |  |  |  |
|  |  |  |  |  |  | 23 | 24h |  | 90.0 |  |  |  |
|  |  |  |  |  |  | 37 | 6h |  | 95.7 |  |  |  |

EMIT= Enzyme multiplied immunoassay technique; HPLC= high performance liquid chromatography; IP=intraperitoneal; PVC= polyvinyl chloride

| **Drug** | **Dosing recommendations on ISPD guidelines for IP route** | **Author/Year** | **Concentration (mg/L)** | **PD Solution** | **PD container material** | **Temperature**  **(°C)** | **Stability in Days (d)/Hours (h)** | **Assay** | **Outcomes** | | | |
| --- | --- | --- | --- | --- | --- | --- | --- | --- | --- | --- | --- | --- |
|  |  |  |  |  |  |  |  |  | **Chemical Stability**  **(Initial concentration remained/%)** | **Antimicrobial activity (%)** | **Physical Stability** | **Microbial Stability** |
| Ceftolozane/  Tazobactam (C/T) | N/A | Harmanjeet *et al*.^(12)^/2020 | 40/20 ^[[6]](#footnote-6)^ | Balance 1.3% glucose | Polyolefine | 4 | 7d | HPLC ^c^ | 99.17/100.51 | Not studied | No colour change examined under white fluorescent light and against white/black background and precipitation using light microscopy method. | Not studied |
|  |  |  | 40/20 ^f^ |  |  | 25 | 6h |  | 100.75/101.69 |  |  |  |
|  |  |  | 20/10 ^[[7]](#footnote-7)^ |  |  | 37 | 12h |  | 100.06/100.06 |  |  |  |
|  |  |  | 40/20 ^f^ | Balance 2.3% glucose | Polyolefine | 4 | 7d |  | 98.21/99.76 |  |  |  |
|  |  |  | 40/20 ^f^ |  |  | 25 | 6h |  | 99.59/99.52 |  |  |  |
|  |  |  | 20/10 ^g^ |  |  | 37 | 12h |  | 99.03/99.75 |  |  |  |
|  |  |  | 20/10 | Dianeal 1.5% glucose | PVC | 4 | 7d |  | 98.28/99.87 |  |  |  |
|  |  |  |  |  |  | 25 | 6h |  | 99.61/98.63 |  |  |  |
|  |  |  |  |  |  | 37 | 12h |  | 100.78/99.79 |  |  |  |
|  |  |  | 20/10 | Dianeal 2.5% glucose | PVC | 4 | 7d |  | 99.23/102.03 |  |  |  |
|  |  |  |  |  |  | 25 | 6h |  | 98.67/100.74 |  |  |  |
|  |  |  |  |  |  | 37 | 12h |  | 97.32/101.15 |  |  |  |
|  |  |  | 20/10 | Dianeal 4.25% glucose | PVC | 4 | 7d |  | 99.21/99.98 |  |  |  |
|  |  |  |  |  |  | 25 | 6h |  | 100.56/99.80 |  |  |  |
|  |  |  |  |  |  | 37 | 12h |  | 100.49/99.89 |  |  |  |
|  |  |  | 20/10 | Extraneal 7.5% icodextrin | PVC | 4 | 7d |  | 97.93/100.27 |  |  |  |
|  |  |  |  |  |  | 25 | 6h |  | 99.76/99.55 |  |  |  |
|  |  |  |  |  |  | 37 | 12h |  | 99.86/99.37 |  |  |  |
|  |  |  | 55.1/27.5 ^[[8]](#footnote-8)^ | Physioneal 1.36% glucose | PVC | 4 | 7d |  | 99.17/99.36 |  |  |  |
|  |  |  | 55.1/27.5 ^h^ |  |  | 25 | 6h |  | 102.45/100.36 |  |  |  |
|  |  |  | 20/10 ^[[9]](#footnote-9)^ |  |  | 37 | 12h |  | 99.31/99.53 |  |  |  |
|  |  |  | 55.1/27.5 ^h^ | Physioneal 2.27% glucose | PVC | 4 | 7d |  | 99.96/99.94 |  |  |  |
|  |  |  | 55.1/27.5 ^h^ |  |  | 25 | 6h |  | 100.29/101.25 |  |  |  |
|  |  |  | 20/10 ^i^ |  |  | 37 | 12h |  | 98.01/99.23 |  |  |  |
|  |  |  | 55.1/27.5 ^h^ | Physioneal 3.86% glucose | PVC | 4 | 7d |  | 98.68/99.27 |  |  |  |
|  |  |  | 55.1/27.5 ^h^ |  |  | 25 | 6h |  | 101.75/99.64 |  |  |  |
|  |  |  | 20/10 ^i^ |  |  | 37 | 12h |  | 97.89/98.56 |  |  |  |

IP=intraperitoneal; HPLC= high performance liquid chromatography; PVC= polyvinyl chloride

| **Drug** | **Dosing recommendations on ISPD guidelines for IP route** | **Author/Year** | **Concentration (mg/L)** | **PD Solution** | **PD container material** | **Temperature**  **(°C)** | **Stability in Days (d)/Hours (h)** | **Assay** | **Outcomes** | | | |
| --- | --- | --- | --- | --- | --- | --- | --- | --- | --- | --- | --- | --- |
|  |  |  |  |  |  |  |  |  | **Chemical Stability**  **(Initial concentration remained/%)** | **Antimicrobial activity (%)** | **Physical Stability** | **Microbial Stability** |
| Clindamycin | **Continuous dosing**:  Maintenance dose:  600 mg in 2L bag (300mg/L) | Kohoe *et al*.^(13)^ /1988 | 200 ^[[10]](#footnote-10)^ | Dianeal PD-2 d | PVC | 8 | 4d | EMIT | 106.3 | Not studied | No visual changes to the colour or precipitation | Not studied |
|  |  |  |  |  |  | 23 | 4d |  | 98.9 |  |  |  |
|  |  | Sewell *et al*.^(8)^/1983 | 10 | Dianeal PD-2 e^,^d | PVC | 25 | 2d | Standard disk diffusion assay with *Bacillus subtilis* | Not studied | 108 | Not studied | Not studied |
|  |  | Tran *et al.*^(14)^/2012 | 150 | Dianeal PD-4 ^d^ | PVC | 37 | 6h | HPLC ^c^ | 100 | Not studied | Not studied | Not studied |
| Cotrimoxazole  (Trimethoprim/  Sulphamethoxazole) | N/A | Holmes and Aldous ^(15)^/1990 | 20/100 | Dianeal PD-2 4.25% glucose | PVC | 20 | 12h | HPLC ^c^ | 95.8/90.7 | Not studied | Not studied | Not studied |
|  |  |  |  |  | Glass ampoule | 20 | 24h |  | 97.5/94.7 |  |  |  |

EMIT= Enzyme multiplied immunoassay technique; IP=intraperitoneal; HPLC= high performance liquid chromatography; PVC= polyvinyl chloride

| **Drug** | **Dosing recommendations on ISPD guidelines for IP route** | **Author/Year** | **Concentration (mg/L)** | **PD Solution** | **PD container material** | **Temperature**  **(°C)** | **Stability in Days (d)/Hours (h)** | **Assay** | **Outcomes** | | | |
| --- | --- | --- | --- | --- | --- | --- | --- | --- | --- | --- | --- | --- |
|  |  |  |  |  |  |  |  |  | **Chemical Stability**  **(Initial concentration remained/%)** | **Antimicrobial activity (%)** | **Physical Stability** | **Microbial Stability** |
| Daptomycin (Cubicin ®) | **Intermittent dosing:**  300 mg in 2L bag/day  (150mg/L)^(16)^  **Continuous dosing:**  Loading dose:  100 mg/L^(17, 18)^, Maintenance dose:  20 mg/L^(17, 18)^ | Parra *et al*.^(19)^/2013 | 20 | Physioneal 35 1.36% glucose ^[[11]](#footnote-11)^ | PVC | 25 | 24h | HPLC | 92.2 | Not studied | No visual changes to the colour or precipitation | Not studied |
|  |  |  |  |  |  | 37 | 6h |  | 92.9 |  |  |  |
|  |  |  |  | Physioneal 35 2.27% glucose ^k^ |  | 25 | 24h |  | 98.0 |  |  |  |
|  |  |  |  |  |  | 37 | 6h |  | 97.9 |  |  |  |
|  |  | Peyro-Saint-Paul *et al*.^(20)^/ 2011 | 50 | Physioneal 40 1.36% glucose ^[[12]](#footnote-12)^ | PVC | 4 | 3d | HPLC | 94.0 | Not studied | No visual changes to the colour or precipitation | Not studied |
|  |  |  |  |  |  | 25 | 24h |  | 96.0 |  |  |  |
|  |  |  |  |  |  | 37 | 6h |  | 102.0 |  |  |  |
|  |  |  |  | Nutrineal |  | 4 | 7d |  | 106.0 |  |  |  |
|  |  |  |  |  |  | 25 | 7d |  | 106.0 |  |  |  |
|  |  |  |  |  |  | 37 | 24h |  | 92.0 |  |  |  |
|  |  |  | 100 | Physioneal 40 1.36% glucose ^l^ | Glass | 4 | 7d |  | 90.0 |  |  |  |
|  |  |  |  |  |  | 25 | 24h |  | 96.0 |  |  |  |
|  |  |  |  |  |  | 37 | 12h |  | 91.0 |  |  |  |
|  |  |  |  | Nutrineal |  | 4 | 7d |  | 109.0 |  |  |  |
|  |  |  |  |  |  | 25 | 7d |  | 97.0 |  |  |  |
|  |  |  |  |  |  | 37 | 6h |  | 92.0 |  |  |  |
|  |  |  |  | Physioneal 40 1.36% glucose ^l^ | PVC | 4 | Not studied |  | Not studied |  |  |  |
|  |  |  |  |  |  | 25 | 6h |  | 93.0 |  |  |  |
|  |  |  |  |  |  | 37 | Not studied |  | Not studied |  |  |  |
|  |  |  |  | Nutrineal |  | 4 | Not studied |  | Not studied |  |  |  |
|  |  |  |  |  |  | 25 | 7d |  | 99.0 |  |  |  |
|  |  |  |  |  |  | 37 | Not studied |  | Not studied |  |  |  |
|  |  |  | 200 | Physioneal 40 1.36% glucose ^l^ | PVC | 4 | 24h |  | 99.0 |  |  |  |
|  |  |  |  |  |  | 25 | 2d |  | 90.0 |  |  |  |
|  |  |  |  |  |  | 37 | 6h |  | 102.0 |  |  |  |
|  |  |  |  | Nutrineal |  | 4 | 7d |  | 108.0 |  |  |  |
|  |  |  |  |  |  | 25 | 7d |  | 93.0 |  |  |  |
|  |  |  |  |  |  | 37 | 6h |  | 91.0 |  |  |  |
|  |  | Ramdas et al.^(21)^/2016 | 25^[[13]](#footnote-13)^ | Balance 1.5% glucose | Polyolefine | 4 | 5d | HPLC ^c^ | >95 | Not studied | No precipitation was observed, but the solution became pale yellowish after 48 hours at 37°C and 7 days at 25°C | Not studied |
|  |  |  |  |  |  | 25 | 3d |  | >90 |  |  |  |
|  |  |  | 20^[[14]](#footnote-14)^ |  |  | 37 | 12h |  | >90 |  |  |  |

IP=intraperitoneal; HPLC= high performance liquid chromatography; PVC= polyvinyl chloride

| **Drug** | **Dosing recommendations on ISPD guidelines for IP route** | **Author/Year** | **Concentration (mg/L)** | **PD Solution** | **PD container material** | **Temperature**  **(°C)** | **Stability in Days (d)/Hours (h)** | **Assay** | **Outcomes** | | | |
| --- | --- | --- | --- | --- | --- | --- | --- | --- | --- | --- | --- | --- |
|  |  |  |  |  |  |  |  |  | **Chemical Stability**  **(Initial concentration remained/%)** | **Antimicrobial activity (%)** | **Physical Stability** | **Microbial Stability** |
| Erythromycin lactobionate | N/A | Kane *et al*.^(22)^/1994 | 150 | Dianeal PD-1 1.5% glucose | PVC | 4 | 2d | HPLC | 98.3 | Not studied | Not studied | Not studied |
|  |  |  |  |  |  | 25 | 3d |  | 97.8 |  |  |  |
|  |  |  |  |  |  | 37 | 8h |  | 91.6 |  |  |  |
|  |  |  |  | Dianeal PD-1 4.25% glucose |  | 4 | 14d |  | 93.7 |  |  |  |
|  |  |  |  |  |  | 25 | 3d |  | 94.0 |  |  |  |
|  |  |  |  |  |  | 37 | 2d |  | 90.6 |  |  |  |
| Fosfomycin | **Intermittent dosing**:  4000 mg/2L bag/day ^(23, 24)^  (=~2000mg/L) | Kussmann *et al*.^(24)^/2017 | 1980 | Extraneal 7.5% icodextrin | PVC | 6 | 14d | LC-MS/disk diffusion inhibition assay with *Escherichia coli* | 94.1 | 97.5 | No visual changes to the colour or precipitation | No microbial contamination was detected using tryptone soya broth |
|  |  |  |  |  |  | 25 | 14d |  | 100.2 | 100.0 |  |  |
|  |  |  |  |  |  | 37 | 24h |  | 93.8 | 101.7 |  |  |
|  |  |  | 1587 | Nutrineal | PVC | 6 | 14d |  | 101.5 | 103.4 |  |  |
|  |  |  |  |  |  | 25 | 14d |  | 95.0 | 100.0 |  |  |
|  |  |  |  |  |  | 37 | 24h |  | 96.7 | 103.4 |  |  |
|  |  |  | 5369 | Physioneal 40 1.36% glucose ^[[15]](#footnote-15)^ (unmixed) | PVC | 6 | 14d |  | 95.3 | 103.1 |  |  |
|  |  |  |  |  |  | 25 | 14d |  | 99.8 | 98.6 |  |  |
|  |  |  | 1980 | Physioneal 1.36% glucose (mixed) ^[[16]](#footnote-16)^ | PVC | 37 | 24h |  | 99.5 | 101.7 |  |  |
|  |  |  | 5369 | Physioneal 40 2.27% glucose (unmixed) ^o^ | PVC | 6 | 14d |  | 97.5 | 100.7 |  |  |
|  |  |  |  |  |  | 25 | 14d |  | 99.1 | 102.3 |  |  |
|  |  |  | 1980 | Physioneal 40 2.27% glucose (mixed) ^p^ | PVC | 37 | 24h |  | 97.2 | 100.8 |  |  |

IP=intraperitoneal; HPLC= high-performance liquid chromatography; LC-MS= High-performance liquid chromatography coupled to a mass spectrometer; PVC= polyvinyl chloride

| **Drug** | **Dosing recommendations on ISPD guidelines for IP route** | **Author/Year** | **Concentration (mg/L)** | **PD Solution** | **PD container material** | **Temperature**  **(°C)** | **Stability in Days (d)/Hours (h)** | **Assay** | **Outcomes** | | | |
| --- | --- | --- | --- | --- | --- | --- | --- | --- | --- | --- | --- | --- |
|  |  |  |  |  |  |  |  |  | **Chemical Stability**  **(Initial concentration remained/%)** | **Antimicrobial activity (%)** | **Physical Stability** | **Microbial Stability** |
| Linezolid | N/A | Manley *et al*.^(25)^/2002 | 150 | Dianeal PD-2 1.5% glucose | PVC | 4 | 7d | HPLC | 107.1 | Not studied | No visual changes to the colour or precipitation | Not studied |
|  |  |  |  |  |  | 25 | 7d |  | 99.0 |  |  |  |
|  |  |  |  |  |  | 37 | 24h |  | 99.1 |  |  |  |
|  |  |  |  | Dianeal PD-2 4.25% glucose | PVC | 4 | 7d |  | 97.2 |  |  |  |
|  |  |  |  |  |  | 25 | 7d |  | 102.4 |  |  |  |
|  |  |  |  |  |  | 37 | 24h |  | 96.1 |  |  |  |
|  |  |  | 300 | Dianeal PD-2 1.5% glucose | PVC | 4 | 7d |  | 99.4 |  |  |  |
|  |  |  |  |  |  | 25 | 7d |  | 100.7 |  |  |  |
|  |  |  |  |  |  | 37 | 24h |  | 102.3 |  |  |  |
|  |  |  |  | Dianeal PD-2 4.25% glucose | PVC | 4 | 7d |  | 101.7 |  |  |  |
|  |  |  |  |  |  | 25 | 7d |  | 98.7 |  |  |  |
|  |  |  |  |  |  | 37 | 24h |  | 98.3 |  |  |  |
|  |  |  | 600 | Dianeal PD-2 1.5% glucose | PVC | 4 | 7d |  | 100.7 |  |  |  |
|  |  |  |  |  |  | 25 | 7d |  | 99.0 |  |  |  |
|  |  |  |  |  |  | 37 | 24h |  | 100.4 |  |  |  |
|  |  |  |  | Dianeal PD-2 4.25% glucose | PVC | 4 | 7d |  | 95.2 |  |  |  |
|  |  |  |  |  |  | 25 | 7d |  | 96.5 |  |  |  |
|  |  |  |  |  |  | 37 | 24h |  | 100.2 |  |  |  |
|  |  | Poeppl *et al*.^(26)^/2018 | 260 | Extraneal 7.5% icodextrin | PVC | 6 | 14d | HPLC & *Bacillus subtilis* inhibition assay | 99.8 | 94.3 | No visual changes to the colour or precipitation | Not studied |
|  |  |  |  |  |  | 25 | 14d |  | 100.7 | 103.8 |  |  |
|  |  |  |  |  |  | 37 | 24h |  | 100.3 | 102.0 |  |  |
|  |  |  | 214 | Nutrineal | PVC | 6 | 14d |  | 100.1 | 100.7 |  |  |
|  |  |  |  |  |  | 25 | 14d |  | 100.6 | 98.8 |  |  |
|  |  |  |  |  |  | 37 | 24h |  | 99.6 | 101.5 |  |  |
|  |  |  | 585 ^[[17]](#footnote-17)^ | Physioneal 40 1.36% glucose | PVC | 6 | 14d |  | 102.0 | 90.1 |  |  |
|  |  |  | 585 ^q^ |  |  | 25 | 14d |  | 100.4 | 103.0 |  |  |
|  |  |  | 260 ^[[18]](#footnote-18)^ |  |  | 37 | 24h |  | 99.7 | 105.6 |  |  |
|  |  |  | 585 ^q^ | Physioneal 40 2.27% glucose | PVC | 6 | 14d |  | 99.8 | 102.5 |  |  |
|  |  |  | 585 ^q^ |  |  | 25 | 14d |  | 100.8 | 101.1 |  |  |
|  |  |  | 260 ^r^ |  |  | 37 | 24h |  | 100.1 | 99.1 |  |  |
| Moxifloxacin | N/A | Fernandez-Varon *et al*.^(27)^/2006 | 25 | Dianeal PD-1 1.36% glucose | PVC | 4 | 14d | HPLC | 98.4 | Not studied | No visual changes to the colour or precipitation | Not studied |
|  |  |  |  |  |  | 25 | 7d |  | 96.6 |  |  |  |
|  |  |  |  |  |  | 37 | 3d |  | 90.1 |  |  |  |
|  |  |  |  | Dianeal PD-1 3.86% glucose | PVC | 4 | 14d |  | 90.7 |  |  |  |
|  |  |  |  |  |  | 25 | 3d |  | 91.2 |  |  |  |
|  |  |  |  |  |  | 37 | 12h |  | 92.8 |  |  |  |

IP=intraperitoneal; HPLC= high performance liquid chromatography; PVC= polyvinyl chloride

| **Drug** | **Dosing recommendations on ISPD guidelines for IP route** | **Author/Year** | **Concentration (mg/L)** | **PD Solution** | **PD container material** | **Temperature**  **(°C)** | **Stability in Days (d)/Hours (h)** | **Assay** | **Outcomes** | | | |
| --- | --- | --- | --- | --- | --- | --- | --- | --- | --- | --- | --- | --- |
|  |  |  |  |  |  |  |  |  | **Chemical Stability**  **(Initial concentration remained/%)** | **Antimicrobial activity (%)** | **Physical Stability** | **Microbial Stability** |
| Ofloxacin | **Continuous dosing:**  Loading dose:  200 mg/L  Maintenance dose:  25 mg/L | Battista *et al*.^(28)^/1995 | 25 | Dianeal PD-1 1.5% glucose | PVC | 4 | 14d | HPLC | 99.1 | Not studied | No visual changes to the colour or precipitation | Not studied |
|  |  |  |  |  |  | 25 | 7d |  | 99.6 |  |  |  |
|  |  |  |  |  |  | 37 | 2d |  | 101.9 |  |  |  |
|  |  |  |  | Dianeal PD-1 4.25% glucose | PVC | 4 | 14d |  | 100.8 |  |  |  |
|  |  |  |  |  |  | 25 | 7d |  | 97.7 |  |  |  |
|  |  |  |  |  |  | 37 | 2d |  | 97.7 |  |  |  |
| Teicoplanin | **Intermittent dosing:**  15 mg/kg every 5 days ^(29, 30)^  **Continuous dosing:**  Loading dose:  400 mg^*^  Maintenance dose:  20 mg/L^(31, 32)^  ^*^_Pharmacokinetic study conducted in intravenous route_^(32)^_. Therefore, drug concentration in PD bag is unknown._ | Manduru *et al*.^(33)^/1996 | 25 | Dianeal PD-2 1.5% glucose | PVC | 25°C for 24h followed by 8h at 37°C | | HPLC ^c^ | 101.1 | Not studied | No visual changes to the colour or precipitation | Not studied |
|  |  |  |  |  |  | 4°C for 7d followed by 16h at 25°C and 8h at 37°C | |  | 92.8 |  |  |  |
| Tigecycline | N/A | Robiyanto *et al*.^(34)^/2015 | 2 | Dianeal 1.5% glucose | PVC | 4 | 14d | HPLC ^c^ | 95.5 | Not studied | No visual changes to the colour or precipitation | Not studied |
|  |  |  |  |  |  | 25 | 3d |  | 93.3 |  |  |  |
|  |  |  |  |  |  | 37 | 12h |  | 95.9 |  |  |  |
|  |  |  |  | Extraneal 7.5% icodextrin | PVC | 4 | 14d |  | 95.8 |  |  |  |
|  |  |  |  |  |  | 25 | 3d |  | 91.1 |  |  |  |
|  |  |  |  |  |  | 37 | 12h |  | 95.6 |  |  |  |
|  |  |  | 4 ^[[19]](#footnote-19)^ | Balance 1.5% glucose | Polyolefine | 4 | 9d |  | 90.0 |  |  |  |
|  |  |  |  |  |  | 25 | 3d |  | 91.8 |  |  |  |
|  |  |  | 2^[[20]](#footnote-20)^ |  |  | 37 | 8h |  | 94.4 |  |  |  |

IP=intraperitoneal; HPLC= high performance liquid chromatography; PVC= polyvinyl chloride

| **Drug** | **Dosing recommendations on ISPD guidelines for IP route** | **Author/Year** | **Concentration (mg/L)** | **PD Solution** | **PD container material** | **Temperature**  **(°C)** | **Stability in Days (d)/Hours (h)** | **Assay** | **Outcomes** | | | |
| --- | --- | --- | --- | --- | --- | --- | --- | --- | --- | --- | --- | --- |
|  |  |  |  |  |  |  |  |  | **Chemical Stability**  **(Initial concentration remained/%)** | **Antimicrobial activity (%)** | **Physical Stability** | **Microbial Stability** |
| Tobramycin | **Intermittent dosing:**  0.6 mg/kg daily^(23)^ | Sewell *et al*.^(8)^/1983 | 10 | Dianeal PD-2 ^d e^ | PVC | 25 | 48h | Standard disk diffusion with *Bacillus subtilis* | Not studied | 107 | Not studied | Not studied |
|  |  | Drake *et al*.^(35)^/1990 | 120 (LD) | Dianeal PD-2 1.5% glucose | PVC | 37 | 8h | Standard disk diffusion bioassay with *Escherichia coli* | Not studied | 99.2 | No visual changes to the colour or precipitation | Not studied |
|  |  |  | 8 (MD) |  |  | 4 | 2d |  |  | 92.9 |  |  |
|  |  |  |  |  |  | 25 | 2d |  |  | 98.7 |  |  |
|  |  | Mason *et al*.^(36)^/1992 | 8 | Dianeal-PD2 2.5% glucose | PVC | 16h @ 25°C + 8h @ 37°C | 24h | Fluorescence polarisation immunoassay ^c^ | 96.5 | Not studied | No visual changes to the colour or precipitation | Not studied |
|  |  | Voges *et al*.^(37)^/2004 | 60 | Physioneal  (mixed) | Clear-Flex ^w^ | 24h @ 25°C + 4h@37°C | <28 | HPLC | 78.9 | Not studied | No visual changes to the colour or precipitation | Not studied |
|  |  |  | 78 ^[[21]](#footnote-21)^ | Physioneal (unmixed)^[[22]](#footnote-22)^ | Clear-Flex ^[[23]](#footnote-23)^ | 25 | 1h |  | 99.1 | Not studied |  |  |
|  |  |  |  |  |  |  | After 1hr ^[[24]](#footnote-24)^ and mixing |  | 92.1 |  |  |  |
|  |  |  | 60 | Nutrineal | Clear-Flex ^w^ | 25 | 24h | Standard disk diffusion bioassay with *Bacillus subtilis* | Not studied | 99.9 |  |  |
|  |  |  |  |  |  | 24h @ 25°C + 4h @ 37°C | 28h |  |  | 101.6 |  |  |
|  |  |  | 60 | Extraneal 7.5% icodextrin | Clear-Flex ^w^ | 25 | 24h | HPLC | 99.0 | Not studied |  |  |
|  |  |  |  |  |  | 24h @ 25°C + 4h @ 37°C | 28h |  | 97.3 |  |  |  |
|  |  |  | 60 | Dianeal PD-4 ^c^ | Clear-Flex ^w^ | 25 | 24h | HPLC | 93.8 |  |  |  |
|  |  |  |  |  |  | 24h @ 25°C + 4h @ 37°C | 28h |  | <90 |  |  |  |
|  |  | Pallota *et al*.^(38)^/2009 | 40 | Extraneal 7.5% icodextrin | PVC | 4 | 14d | HPLC ^c^ | 94.6 | Not studied | No visual changes to the colour or precipitation | Not studied |
|  |  |  |  |  |  | 25 | 7d |  | 90.5 |  |  |  |
|  |  |  |  |  |  | 37 | 24h |  | 90.0 |  |  |  |
|  |  | Deslandes *et al*.^(39)^/2016 | 4 | Physioneal 1.36% glucose ^[[25]](#footnote-25)^ | PVC | 37 | 24h | HPLC | 105 | Not studied | No visual changes to the colour or precipitation | Not studied |
|  |  |  |  | Physioneal 3.86% glucose ^y^ | PVC | 37 | 24h |  | 95 |  |  |  |
|  |  |  |  | Extraneal 7.5% icodextrin | PVC | 12h @ 22°C followed by 12h @ 37°C | 24h |  | 105 |  |  |  |

IP=intraperitoneal; HPLC= High-performance liquid chromatography; PVC= polyvinyl chloride

**Supplementary Table 2:** Compatibility of culture-directed antibiotic with other antibiotics in the PD solutions

| **Drug** | **Author/Year** | **Concentration (mg/L)** | **PD Solution** | **PD container material** | **Temperature**  **(°C)** | **Stability in Days (d)/Hours (h)** | **Assay** | **Outcomes** | | | |
| --- | --- | --- | --- | --- | --- | --- | --- | --- | --- | --- | --- |
|  |  |  |  |  |  |  |  | **Chemical Stability**  **(initial concentration remained/%)** | **Antimicrobial activity (%)** | **Physical Stability** | **Microbial Stability** |
| Ceftazidime/  tobramycin | Mason *et al*.^(40)^/1992 | 125/8 | Dianeal PD-2 2.5% glucose | PVC | 16h @ 25°C + 8h @ 37°C | 24h | Fluorescence polarisation immunoassay ^c^ | 95.0/96.7 | Not studied | No visual changes to the colour or precipitation | Not studied |
| Ceftazidime/  tobramycin | Deslandes *et al*.^(39)^/2006 | 125/4 | Physioneal 1.36% glucose | PVC | 37 | 12.8h | HPLC | 102.6/102.5 | Not studied | No visual changes to the colour or precipitation | Not studied |
|  |  |  | Physioneal 3.86% glucose | PVC | 37 | 6h | HPLC | 101.7/100.0 |  |  |  |
|  |  |  | Extraneal 7.5% icodextrin | PVC | 12h @ 22°C followed by 12h @ 37°C | 24h | HPLC | 103.6/105.0 |  |  |  |
| Ceftazidime/  teicoplanin | Manduru *et al*.^(33)^/1996 | 100/25 | Dianeal PD-2 1.5% glucose | PVC | 7d at 4°C followed by 16h at 25°C and 8h at 37°C | | HPLC ^c^ | 92.7/91.1 | Not studied | No visual changes to the colour or precipitation | Not studied |
| Clindamycin/  gentamicin | Kehoe *et al*.^(13)^/1988 | 200/10 | Dianeal PD-2 ^d^ | PVC | 8 | 4d | EMIT | 100/96.7 | Not studied | No visual changes to the colour or precipitation | Not studied |
|  |  |  |  |  | 23 | 4d | EMIT | 100.0/104.4 |  |  |  |

EMIT= Enzyme multiplied immunoassay technique; HPLC= High-performance liquid chromatography; PVC= polyvinyl chloride

**REFERENCES**

1. Janknegt R, Paulissen A, Hooymans PM, Lohman JJ, Hermens WA. Stability of amphotericin B in CAPD fluid. Peritoneal Dialysis International. 1990;10(4):287-9.

2. Manley HJ, Grabe DW, Norcross M, Cooperman TA, Stinchcomb AL, Hass M, et al. Stability of amphotericin B lipid complex (Abelcet) in peritoneal dialysis solutions. Peritoneal Dialysis International. 2000;20(1):87-90.

3. Tobudic S, Donath O, Vychytil A, Forstner C, Poeppl W, Burgmann H. Stability of anidulafungin in two standard peritoneal dialysis fluids. Peritoneal Dialysis International. 2014;34(7):798-802.

4. Cheng IKP, Chan C-Y, Wong WT. A Randomised Prospective Comparison of Oral Ofloxacin and Intraperitoneal Vancomycin plus Aztreonam in the Treatment of Bacterial Peritonitis Complicating Continuous Ambulatory Peritoneal Dialysis (CAPD). Peritoneal Dialysis International. 1991;11(1):27-30.

5. Tobudic S, Prager I, Kussmann M, Obermüller M, Ursli M, Zeitlinger M, et al. Compatibility of aztreonam in four commercial peritoneal dialysis fluids. Sci Rep. 2020;10(1):1788.

6. Albin HC, Demotes-Mainard FM, Bouchet JL, Vincon GA, Martin-Dupont C. Pharmacokinetics of intravenous and intraperitoneal cefotaxime in chronic ambulatory peritoneal dialysis. Clin Pharmacol Ther. 1985;38(3):285-9.

7. Paap CM, Nahata MC. Stability of cefotaxime in two peritoneal dialysis solutions. American Journal of Hospital Pharmacy. 1990;47(1):147-50.

8. Sewell DL, Golper TA, Brown SD, Nelson E, Knower M, Kimbrough RC. Stability of single and combination antimicrobial agents in various peritoneal dialysates in the presence of insulin and heparin. Am J Kidney Dis. 1983;3(3):209-12.

9. Farooqi S, Naqvi BS, Gauhar S. Stability study of antibiotic (Cefotaxime) in peritoneal dialysis solution with validation of analyzing method. International Journal of Pharmacy and Pharmaceutical Sciences. 2013;5(SUPPL 3):930-4.

10. Albin H, Ragnaud JM, Demotes-Mainard F, Vinçon G, Couzineau M, Wone C. Pharmacokinetics of intravenous and intraperitoneal ceftriaxone in chronic ambulatory peritoneal dialysis. Eur J Clin Pharmacol. 1986;31(4):479-83.

11. Nahata MC. Stability of ceftriaxone sodium in peritoneal dialysis solutions. Dicp. 1991;25(7-8):741-2.

12. Harmanjeet H, Jani H, Zaidi STR, Wanandy T, Castelino RL, Sud K, et al. Stability of ceftolozane and tazobactam in different peritoneal dialysis solutions. Perit Dial Int. 2020;40(5):470-6.

13. Kehoe WA, Weber JN, Fries DS. The Stability and Compatibility of Clindamycin Phosphate and Gentamicin Sulfate Alone and in Combination in Peritoneal Dialysis Solution. Peritoneal Dialysis International. 1988;8(2):153-4.

14. Tran MD, Sharley N, Ward M. Stability of Amoxycillin, Clindamycin and Meropenem in Peritoneal Dialysis Solution. Journal of Pharmacy Practice and Research. 2012;42(3):218-22.

15. Holmes SE, Aldous S. Stability of cotrimoxazole in peritoneal dialysis fluid. Peritoneal Dialysis International. 1990;10(2):157-60.

16. Paul LPS, Ficheux M, Debruyne D, Loilier M, Bouvier N, Morello R, et al. Pharmacokinetics of 300 mg/d Intraperitoneal Daptomycin: New Insight from the DaptoDP Study. Peritoneal Dialysis International. 2018;38(6):463-6.

17. Lin SY, Ho MW, Liu JH, Liu YL, Yeh HC, Hsieh TL, et al. Successful Salvage of Peritoneal Catheter in Unresolved Methicillin-Resistant <i>Staphylococcus aureus</i> Peritonitis by Combination Treatment with Daptomycin and Rifampin. Blood Purification. 2011;32(4):249-52.

18. Huen SC, Hall I, Topal J, Mahnensmith RL, Brewster UC, Abu-Alfa AK. Successful Use of Intraperitoneal Daptomycin in the Treatment of Vancomycin-Resistant Enterococcus Peritonitis. American Journal of Kidney Diseases. 2009;54(3):538-41.

19. Parra MA, Campanero MA, Sádaba B, Irigoyen A, García-López L, Fernandez-Reyes MJ, et al. Effect of glucose concentration on the stability of daptomycin in peritoneal solutions. Perit Dial Int. 2013;33(4):458-61.

20. Peyro Saint Paul L, Albessard F, Gaillard C, Debruyne D, Ryckelynck JP, Coquerel A, et al. Daptomycin compatibility in peritoneal dialysis solutions. Perit Dial Int. 2011;31(4):492-5.

21. Ramdas S, Yousaf F, Shastri MD, Wanandy T, Zaidi STR, Khandagale M, et al. Stability of daptomycin in peritoneal dialysis solutions packaged in dual-compartment infusion bags. Eur J Hosp Pharm. 2016;23(1):57-60.

22. Kane MP, Bailie GR, Moon DG, Siu I, Eisele G. Stability of erythromycin lactobionate in peritoneal dialysate solutions. Peritoneal Dialysis International. 1994;14(1):79-81.

23. Li PK-T, Chow KM, Cho Y, Fan S, Figueiredo AE, Harris T, et al. ISPD peritonitis guideline recommendations: 2022 update on prevention and treatment. Peritoneal Dialysis International. 2022;42(2):110-53.

24. Kussmann M, Baumann A, Hauer S, Pichler P, Zeitlinger M, Wiesholzer M, et al. Compatibility of fosfomycin with different commercial peritoneal dialysis solutions. Eur J Clin Microbiol Infect Dis. 2017;36(11):2237-42.

25. Manley HJ, McClaran ML, Bedenbaugh A, Peloquin CA. Linezolid stability in peritoneal dialysis solutions. Perit Dial Int. 2002;22(3):419-22.

26. Poeppl W, Rainer-Harbach E, Kussmann M, Pichler P, Zeitlinger M, Wiesholzer M, et al. Compatibility of linezolid with commercial peritoneal dialysis solutions. Am J Health Syst Pharm. 2018;75(19):1467-77.

27. Fernández-Varón E, Marín P, Espuny A, Villamayor L, Escudero E, Cárceles C. Stability of moxifloxacin injection in peritoneal dialysis solution bags (Dianeal PD1 1.36% and Dianeal PD1 3.86%). J Clin Pharm Ther. 2006;31(6):641-3.

28. Battista C, Kane MP, Moon DG, Bailie GR. Stability of ofloxacin in peritoneal dialysis solutions. Perit Dial Int. 1995;15(1):72-4.

29. Liakopoulos V, Leivaditis K, Nikitidou O, Divani M, Antoniadi G, Dombros N. Intermittent Intraperitoneal Dose of Teicoplanin in Peritoneal Dialysis–Related Peritonitis. Peritoneal Dialysis International. 2012;32(3):365-6.

30. Schaefer F, Klaus G, MÜLler-Wiefel DE, Mehls O, The Mid-European Pediatric Peritoneal Dialysis Study G. IntermittentversusContinuous Intraperitoneal Glycopeptide/Ceftazidime Treatment in Children with Peritoneal Dialysis-Associated Peritonitis. Journal of the American Society of Nephrology. 1999;10(1).

31. Lupo A, Rugiu C, Bernich P, Laudon A, Marcantoni C, Mosconi G, et al. A prospective, randomized trial of two antibiotic regimens in the treatment of peritonitis in CAPD patients: teicoplanin plus tobramycin versus cephalothin plus tobramycin. Journal of Antimicrobial Chemotherapy. 1997;40(5):729-32.

32. Finch RC, Holliday AP, Innes A, Burden RP, Morgan AG, Shaw PN, et al. Pharmacokinetic behavior of intraperitoneal teicoplanin during treatment of peritonitis complicating continuous ambulatory peritoneal dialysis. Antimicrob Agents Chemother. 1996;40(8):1971-2.

33. Manduru M, Fariello A, White RL, Fox JL, Bosso JA. Stability of ceftazidime sodium and teicoplanin sodium in a peritoneal dialysis solution. Am J Health Syst Pharm. 1996;53(22):2731-4.

34. Robiyanto R, Zaidi ST, Shastri MD, Castelino RL, Wanandy ST, Jose MD, et al. Stability of Tigecycline in Different Types of Peritoneal Dialysis Solutions. Perit Dial Int. 2016;36(4):410-4.

35. Drake JM, Myre SA, Staneck JL, Draeger RW. Antimicrobial activity of vancomycin, gentamicin, and tobramycin in peritoneal dialysis solution. Am J Hosp Pharm. 1990;47(7):1604-6.

36. Mason NA, Johnson CE, O'Brien MA. Stability of ceftazidime and tobramycin sulfate in peritoneal dialysis solution. American Journal of Hospital Pharmacy. 1992;49(5):1139-42.

37. Voges M, Faict D, Lechien G, Taminne M. Stability of drug additives in peritoneal dialysis solutions in a new container. Perit Dial Int. 2004;24(6):590-5.

38. Pallotta KE, Elwell RJ, Nornoo AO, Manley HJ. Stability of tobramycin and ceftazidime in icodextrin peritoneal dialysis solution. Perit Dial Int. 2009;29(1):52-7.

39. Deslandes G, Grégoire M, Bouquié R, Le Marec A, Allard S, Dailly E, et al. Stability and Compatibility of Antibiotics in Peritoneal Dialysis Solutions Applied to Automated Peritoneal Dialysis in The Pediatric Population. Perit Dial Int. 2016;36(6):676-9.

40. Mason NA, Johnson GE, O'Brien MA. Stability of ceftazidime and tobramycin sulfate in peritoneal dialysis solution. American Journal of Hospital Pharmacy. 1992;49(5):1139-42.

1. Aztreonam was added to the glucose compartment of the Physioneal PD solution and remained separated from the non-glucose compartment when stored at refrigeration and room temperature. [↑](#footnote-ref-1)
2. At 37°C, the author first injected the aztreonam into the glucose compartment of the Physioneal PD solution. The glucose and non-glucose compartments were then together immediately. [↑](#footnote-ref-2)
3. Stability-indicating [↑](#footnote-ref-3)
4. The authors did not specify the glucose concentrations in the PD solutions. [↑](#footnote-ref-4)
5. Contained heparin 500 units per litre. [↑](#footnote-ref-5)
6. *Balance 1.3% & 2.3% glucose*: C/T was added to the non-glucose compartment and kept separated from the glucose compartment throughout the storage period at refrigeration and room temperature. Final drug concentration after mixing: C/T 20/10mg/L [↑](#footnote-ref-6)
7. *Balance 1.3% & 2.3% glucose*: C/T concentration after mixing solutions from the two-compartments together (glucose and non-glucose) immediately before warming to 37°C on a warming plate. [↑](#footnote-ref-7)
8. *Physioneal 1.36%, 2.27% & 3.86%:* C/T was added to the glucose compartment and remained separated from the non-glucose compartment throughout the storage period at refrigeration and room temperature. Final drug concentration after mixing: C/T 20/10mg/L [↑](#footnote-ref-8)
9. C/T concentration after mixing solutions from the two-compartments together (glucose and lactate/bicarbonate solution) in Physioneal PD solutions immediately before warming to 37°C on a warming plate. [↑](#footnote-ref-9)
10. Authors reported that final drug concentrations could be lower due to an approximately 4% overfill of the bags [↑](#footnote-ref-10)
11. Daptomycin was added to the glucose-compartment of the Physioneal PD solutions, followed by mixing the solutions (glucose and non-glucose solutions) from two compartments immediately and then stored at room and body temperature. [↑](#footnote-ref-11)
12. Daptomycin was added in the glucose-compartment of the Physioneal PD solutions. However, the author did not specify whether the stability test was conducted before or after mixing solutions from the 2 compartments throughout the storage period. [↑](#footnote-ref-12)
13. Daptomycin was added in non-glucose compartment (1.25L) and stored separated at 4°C and 25°C. The solution from two compartments are mixed together after daptomycin is added into the non-glucose compartment to obtain final concentration of daptomycin 20 mg/L and stored at 37°C. [↑](#footnote-ref-13)
14. The solution from two compartments was mixed together after the addition of daptomycin to the non-glucose compartment to obtain final concentration of daptomycin 20 mg/L and stored at 37°C. [↑](#footnote-ref-14)
15. Fosfomycin was added in the glucose-compartment and remained separated from the non-glucose compartment when stored at refrigeration and room temperature. [↑](#footnote-ref-15)
16. Two compartments were mixed immediately after fosfomycin was added into the PD bag. [↑](#footnote-ref-16)
17. Physioneal PD solutions: Linezolid was added into the glucose-compartment and remained separated from the non-glucose compartment remained throughout the storage duration at refrigeration and room temperature. [↑](#footnote-ref-17)
18. Physioneal PD solutions: The glucose and non-glucose compartments were mixed immediately after linezolid was added into the glucose-compartment. [↑](#footnote-ref-18)
19. Balance 1.5% glucose solution: Tigecycline 4 mg/L was administered in the non-glucose compartment (1.25L) and remained separated on refrigeration and room temperature. To obtain final concentration of 2 mg/L after mixing solutions from the non-glucose and glucose compartments (total 2.5L). [↑](#footnote-ref-19)
20. Balance 1.5% glucose solution: At body temperature, tigecycline was added into the non-glucose compartment and then mixed immediately before warming. [↑](#footnote-ref-20)
21. Tobramycin 78 mg/L (33% increased of initial drug concentration to achieve comparable final drug concentrations (60 mg/L) after mixing two compartments) [↑](#footnote-ref-21)
22. Stability was evaluated in four phases: just after the addition, 15 and 60 minutes of the tobramycin into the glucose compartment, and just after mixing of the two compartments. [↑](#footnote-ref-22)
23. Composed of five co-extruded layers of different polyolefins [↑](#footnote-ref-23)
24. Stability of the unmixed Physioneal solution was tested 60 minutes after tobramycin was added into the glucose compartment, and just after mixing the glucose and non-glucose compartment together. [↑](#footnote-ref-24)
25. Physioneal 1.36% glucose solutions: Tobramycin was first added in the glucose-compartment. The glucose and non-glucose compartments were then mixed immediately after tobramycin was added. [↑](#footnote-ref-25)
